# Role of Dual-Redox Couples in Antiperovskite Li_2_FeSeO Cathodes

**DOI:** 10.1021/acs.chemmater.5c01341

**Published:** 2025-11-11

**Authors:** Tian Dai, Heesoo Park, Anders Brennhagen, Niels Højmark Andersen, Marco Giorgetti, Martin Valldor, Alexey Y. Koposov

**Affiliations:** † Centre for Material Science and Nanotechnology, Department of Chemistry, 6305 University of Oslo, P.O. Box 1033, Blindern, 0371 Oslo, Norway; ‡ Department of Chemistry, University of Oslo, P.O.Box 1033, Blindern, 0315 Oslo, Norway; § Department of Industrial Chemistry, 9296 University of Bologna, Campus Navile, Via Piero Gobetti 85, Bologna 40139, Italy

## Abstract

In recent years, cathode materials for Li-ion batteries with antiperovskite (AP) structure have attracted growing interest due to their high capacity, chemical flexibility, low cost, and relatively simple synthesis. Among AP cathodes, Li_2_FeSeO has emerged as a promising candidate, demonstrating high capacity and good cycling stability. However, the lack of fundamental knowledge of the operating mechanism of AP cathodes significantly impedes efforts for further improvement. In this work, the redox mechanism and structural evolution of Li_2_FeSeO cathode material during electrochemical cycling are revealed through a combination of *operando* and *ex situ* X-ray diffraction, X-ray absorption spectroscopy, and Raman spectroscopy coupled with density functional theory calculations. Within the voltage range of 1–3 V *vs* Li/Li^+^, two stages in the (de)­lithiation process during the initial cycle are determined, involving simultaneous redox activity of Se^2–^ and Fe^2+^ with strong local structural distortion, leading to the formation of Se_2_^2–^ and Fe^3+^ species in the delithiated state and possible formation of Fe^0^ in the lithiated state. While the dual-redox activity of Se^2–^ and Fe^2+^ ions is the key to high capacity, the −O–Fe–O– framework serves as a crucial element for reversibility during cycling. The study highlights the potential for optimizing the structural frameworks of AP cathodes.

## Graphical abstract

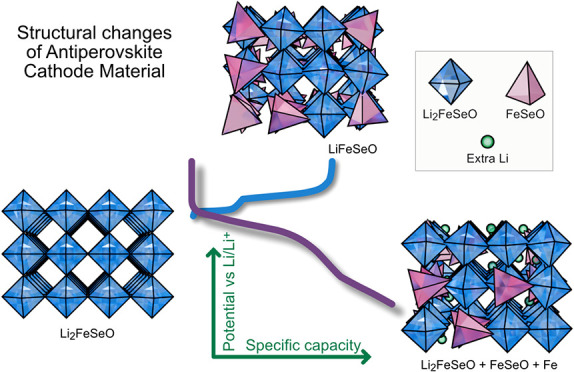

## 1 Introduction

The growing demand for rechargeable Li-ion batteries (LIBs) calls for new sustainable electrode materials. These materials should be obtained through low-cost and scalable approaches while using abundant elements, improving the electrochemical performance and also ensuring compatibility with processing technologies used today.^1,2^ Many components of LIBs could be improved to increase the energy density, with active cathode materials being considered as one of the main limitations.^3^ To date, the majority of research in the area of cathode materials has been focused on improvement of several classes of cathode materials, such as olivine LiFePO_4_ (LFP), layered-oxide LiNi_*x*_Mn_*y*_Co_1‑*x*‑*y*_O_2_ (NMC), and spinel LiMn_2_O_4_ (LMO).^4,5^ Therefore, to overcome the fundamental constraints of their structural characteristics, it is essential to explore alternative cathode materials that may drive advancements toward cost-effective, environmentally sustainable, and intrinsically safe battery solutions.

A class of compounds with an antiperovskite (AP) structure and a general chemical formula of Li_2_ *TMCh*O (*TM* = transition metals: Mn^2+^, Fe^2+^,...; *Ch* = chalcogenides: S^2–^ and Se^2–^), has recently been discovered as promising cathode materials for LIB application.^6–8^ These materials possess a cubic unit cell structure (space group: *Pm*3̅*m*), where *TM* ^2+^ and Li^+^ share the cubic face-centered positions, resulting in a cation-disordered arrangement which greatly benefits the ionic transport of Li^+^. APs have attracted considerable attention for several reasons: the theoretical capacity of Li_2_ *TMCh*O estimated from approximately 230 mAh g^–1^ to 460 mAh g^–1^ (when *TM* = Fe^2+^; *Ch* = S^2–^) depending on assumed extraction of one or two Li^+^ per formula unit. In the first cycle, Li_2_FeSeO can accommodate additional Li^+^ during lithiation compared to delithiation, due to structural flexibility within the AP framework.^6,9,10^ In addition, as Li^+^ and some *TM* ^2+^ ions (Cr^2+^, Mn^2+^, Fe^2+^, Ni^2+^, *etc*.) have similar ionic radii, a great variety of APs can be prepared using abundant elements, avoiding critical materials such as Co.^11,12^ Such structural flexibility also enables partial substitution of cations and anions, offering means to tune Li^+^ diffusion, adjust operating electrochemical potential, and regulate cation disorder.^13–16^ In addition, it has been demonstrated that the AP cathode materials can be synthesized *via* a one-step solid-state reaction (SSR) or mechanochemical reaction (MC, or ball milling), which are solvent-free and scalable methods that can enable the efficient production of high-purity materials with minimal environmental impact.^7,17^

Building on structural flexibility, significant efforts have been dedicated to enhance the electrochemical performance of APs in terms of capacity and cycling stability. For instance, Gorbunov *et al*. investigated partial cation substitution in Li_2_Fe_1–*x*_ *M_x_* SO (M = Mn^2+^, Co^2+^) and demonstrated that Mn^2+^ substitution enhances structural stability during cycling.^14,15^ Mohamed *et al.* explored anion-substituted Li_2_FeS_1–*x*_Se*_x_* O, improving thermal and moisture stability and highlighting the role of chalcogenide in enhancing capacity retention.^16^

Recent investigations have also sought to elucidate the chemical mechanisms and structural evolution of AP cathode materials during cycling. A few studies have demonstrated a combined cationic and anionic process in APs using cyclic voltammetry (CV), where both transition metals and chalcogenides participate in the redox reaction during (de)­lithiation.^10,18,19^ Mikhailova *et al.* conducted *operando* studies using X-ray diffraction (XRD), X-ray absorption spectroscopy (XAS), and X-ray photoelectron spectroscopy (XPS) on Li_2_FeSO. The proposed mechanism involved oxidation of Fe^2+^ to Fe^3+^ in the low-voltage region during the first charge followed by the oxidation of S^2–^ to elemental sulfur S^0^ at higher voltages.^19^ However, the XAS study was primarily focused on the Fe *K*-edge, as Fe was considered to be the main redox-active element in AP cathodes. Mohamed *et al.* carried out *operando* XAS on both Fe and Se *K*-edges in Li_2_FeS_0.7_Se_0.3_O, but the anionic behavior and local structural evolution upon cycling were not discussed in detail.^16^ Overall, previous *operando* studies confirmed the reversible redox activity of Fe^2+/3+^; however, the high capacity corresponding to the transfer of approximately 2 Li^+^ per formula unit during the initial cycles in both Li_2_FeSO and Li_2_FeSeO should correspond to the electrochemical involvement of anions (chalcogenides), as the formation of a stable Fe^4+^ is unlikely under the choosen potential range of 1–3 V *vs* Li/Li^+^ for AP cathodes. To date, the behavior of chalcogenides remains largely unexplored, leaving the understanding of their working mechanism in its infancy, despite the crucial role of the anion redox process in enhancing the capacity and cycling stability.^16^

In this work, we reveal the redox mechanism and structural evolution of Li_2_FeSeO, exploring the reasons behind its high capacity and chemical stability. To investigate its structural evolution at the atomistic level during cycling, a combination of *operando* and *ex situ* techniques was employed, including XRD, pair distribution function (PDF), XAS, and Raman spectroscopy. Density functional theory (DFT) modeling was carried out to relate intermediate structures upon cycling, providing a comprehensive description of the working mechanism in APs. The thorough investigation reveals a distinct two-stage delithiation process during the initial charge and discharge, which is attributed to the redox activity of both Se^2–^ and Fe^2+^. The revealed cycling mechanism not only clarifies the redox behavior of Li_2_FeSeO but also provides insights applicable to other AP family compositions, thereby expanding the available structure of cathode materials in LIBs.

## 2 Results and Discussion

### 2.1 Structural and Electrochemical Characterizations

Li_2_FeSeO materials were prepared in a powder form by solid-state synthesis using a previously reported procedure.^7^ Li_2_FeSeO has a cation-disordered structure, where 4 Li^+^ and 2 Fe^2+^ ions randomly coshare the 3*c* Wyckoff position of a unit cell. Figure 1a illustrates the atomic structure of Li_2_FeSeO with centering-ion configurations of O^2–^, Se^2–^, and Fe^2+^/Li^+^. The detailed discussion of cation disorder can be found elsewhere.^6,11,20–22^

**Figure 1:**
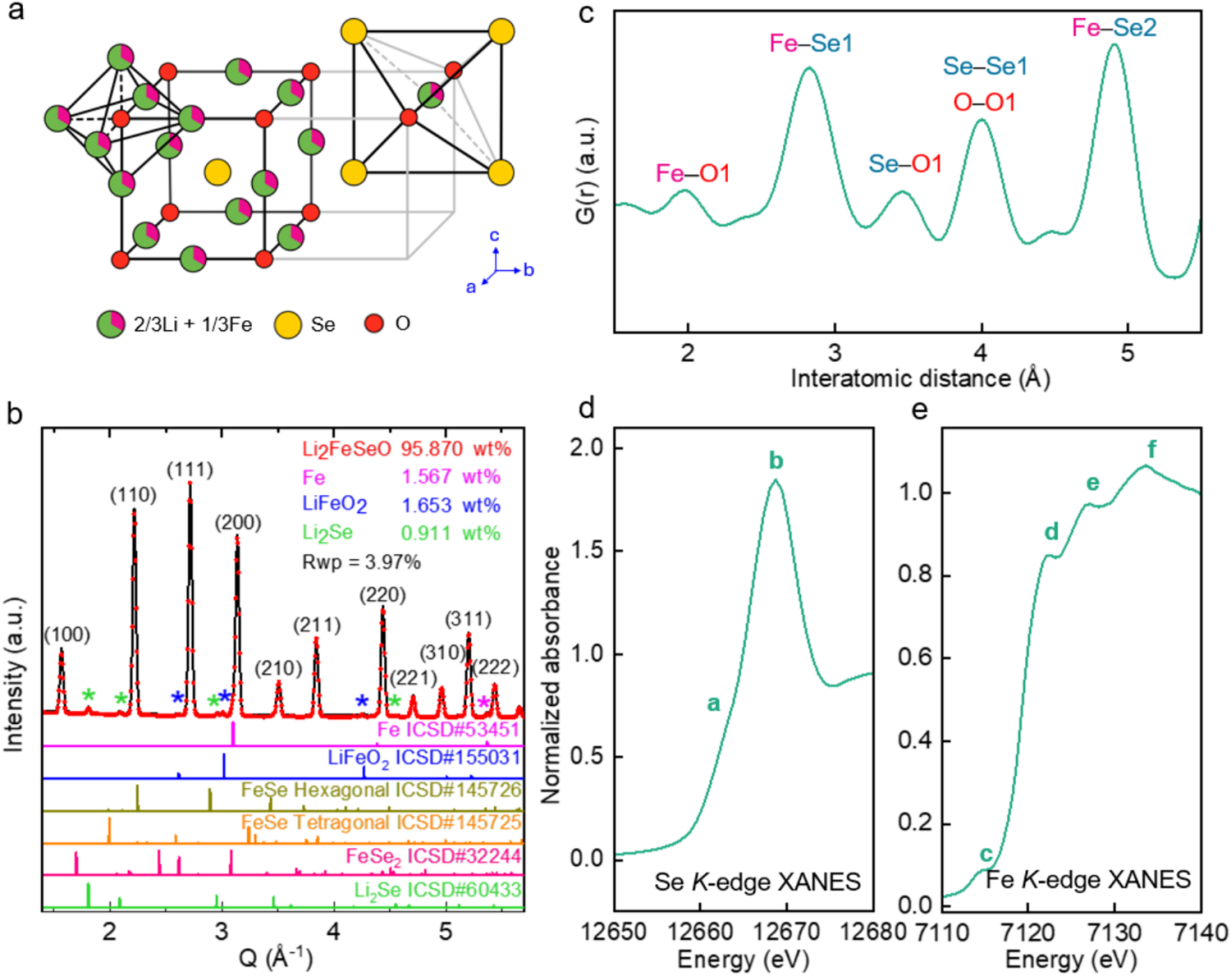
(a) Crystal structure of Li_2_FeSeO showing O^2–^, Se^2–^, and Fe^2+^/Li^+^ as central ions. (b) XRD patterns and the Rietveld refinement results for as-synthesized Li_2_FeSeO. (c) PDF of Li_2_FeSeO. (d) and (e) XANES spectra of Se and Fe *K*-edges, respectively.

Pristine Li_2_FeSeO powder was characterized using XRD, PDF, and XAS. Figure 1b shows the XRD pattern of Li_2_FeSeO with the corresponding Rietveld refinement. This analysis confirmed the presence of AP Li_2_FeSeO as the main phase with a weight percentage of >95%. The lattice parameter was found to be 4.003Å, which corresponds to the previously reported value.^7^ Small amounts (<2%) of Fe, LiFeO_2_, and Li_2_Se were also identified as impurities, but these were not expected to significantly impact the results from the following characterizations. For complete characterization of the local structure, Figure 1c shows the PDF for the same sample with the peak positions labeled according to the corresponding interatomic distances, where number 1 corresponds to the nearest atoms (first coordination shell) and number 2 corresponds to the second nearest atoms (second coordination shell) of each specific element.

Figure 1d shows the Se *K*-edge X-ray absorption near-edge structure (XANES) of Li_2_FeSeO. Se^2–^, being 12-coordinated by 8 Li^+^ and 4 Fe^2+^ randomly distributed on average (Figure 1a), initially exhibits centrosymmetric local coordination. The outer electron shell 4p of Se^2–^ is filled with 6 electrons with no unoccupied site for excited core electrons, giving the rising edge peak ***b*** at 12668.6 eV that corresponds to 1s→5p transition, with no pre-edge feature. However, a small shoulder is observed and is marked as ***a***, the apreance of this shoulder is likely due to asymmetrical coordination induced by local ordering.

Figure 1e shows the Fe *K*-edge XANES spectrum of Li_2_FeSeO. The Fe *K*-edge absorption is dominated by 1s→3d and 1s→4p transitions. The shoulders and peaks in the pristine state are marked as ***c**–**f***. Peak ***c*** is the pre-edge peak corresponding to the 1s→3d transition: Fe has an octahedral coordination environment with 2 apical O^2–^ and 4 square-planar Se^2–^ ions (Figure 1a), a high centrosymmetric motif that inhibits 1s→3d transition; therefore, the weak pre-edge peak ***c*** observed in the pristine sample may arise from local distortions due to *cis*-configuration of the Fe^2+^/Li^+^ arrangement and a potential Jahn–Teller distortion.^23^ The shoulder peak at ***d*** is likely attributed to the dipole-allowed 1s→4p transition *via* a two-step mechanism. Initially, the core electron is excited to the 4p orbital, causing a relative decrease in the metal cation energy levels. This results in charge transfer from the Se 4p orbital to the Fe 3d orbital, leading to a final state of 1s^1^1c^1^2s^2^2p^6^3d^n+1^L4p^1^, where L represents a ligand hole. Peaks ***e*** and ***f*** may correspond to the dipole-allowed Fe 1s→4p transitions mixed with Se p (d) transition.^24–26^

The morphology of the studied materials was also characterized using scanning electron microscopy (SEM), where pristine samples exhibited polycrystalline morphology with particle sizes ranging from 1 to 50 μm featuring cubic or cuboid shapes (Figure S1 in the Supporting Information (SI)).

Li_2_FeSeO-based cathodes were electrochemically evaluated in half-cells with Li foil as a counter electrode by galvanostatic cycling (GC) using the following electrolyte: 1.2 M LiPF_6_ in a 3:7 (vol %) mixture of ethylene carbonate (EC) and ethyl methyl carbonate (EMC), with 2 wt % vinylene carbonate (VC) and 10 wt % fluoroethylene carbonate (FEC) as additives. Details on electrode preparation and coin cell assembly are provided in Sections 4 and S1. The cycling behavior of Li_2_FeSeO electrodes at several current densities ranging from 200 to 2000 mA g^–1^ within a voltage range of 1–3 V *vs* Li/Li^+^ (The voltage value in the text refers to the potential difference between the working electrode and Li/Li^+^ reference, unless specified otherwise) was also evaluated and is shown in Figure 2a. When cycled at 200 mA g^–1^, the material exhibited the initial capacity of ∼290 mAh g^–1^ and retained a higher capacity compared with samples cycled at elevated current densities. Even though the initial capacity slightly decreased at higher current densities, the electrochemical performance is well maintained with capacities above 90 mAh g^–1^ after 300 cycles even at 2000 mA g^–1^. Figure S4 in the SI further presents charge/discharge curves corresponding to different cycling rates.

**Figure 2:**
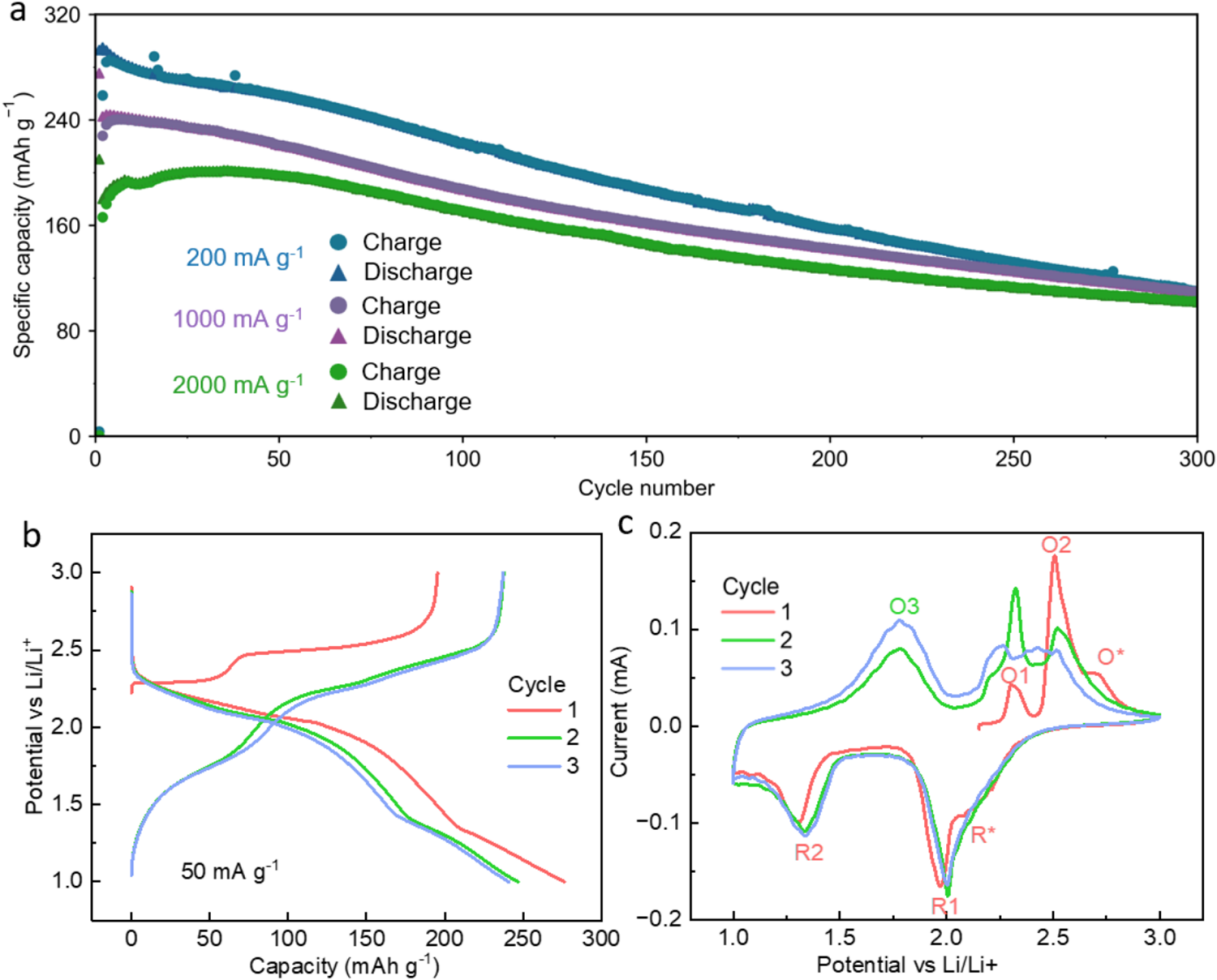
Electrochemical performance of Li_2_FeSeO: (a) capacity per cycle (CPC) at various rates; (b) GC profile at 50 mA g^–1^ for the first three cycles; (c) CV profile for the first three cycles. The voltage range was selected to be 1–3 V *vs* Li/Li^+^.

To better illustrate the cycling behavior in the early stages, the GC curves for the first three cycles are shown in Figure 2b. A measurement protocol with a current density of 50 mA g^–1^ was selected for this figure to provide a better resolution of the GC profile. The first charge resulted in a specific capacity of 196 mAh g^–1^ corresponding to the extraction of approximately 1.2 Li^+^ per formula unit, forming Li_0.8_FeSeO. The first charge exhibits two distinct voltage plateaus, where the first plateau (2.2–2.4 V) contributes to 60 mAh g^–1^ capacity and the second one (2.4–3.0 V) provides additional 136 mAh g^–1^. However, during the subsequent discharge, the capacity increases to approximately 277 mAh g^–1^, where two plateaus in the GC are also observed. The extra capacity during discharge corresponds to the reinsertion of approximately 1.7 Li^+^, leading to a formation of a compound with a possible composition of Li_2.5_FeSeO. This increase in Li^+^ content is associated with a low-voltage process occurring below 2 V. Such differences between the first charge and discharge capacities have been observed in some of the previous studies.^9,18,19^ This apparent “over-lithiation” behavior, along with the limited Li^+^ extraction observed during the first charge, might suggest Li deficiency in the pristine material initially. However, this possibility is unlikely, as previous inductively coupled plasma optical emission spectroscopy (ICP-OES) analyses confirmed the presence of two Li^+^ ions within the AP structure, whereas our synthesis followed the same approach.^7,8^ In addition, previous work has also demonstrated that more than 1.25 Li extraction per formula unit leads to structural collapse.^12^ Therefore, the “over-lithiation” at low voltages is more likely due to the structural flexibility and formation of metallic Fe instead of Li deficiency and will be further discussed in Sections 2.2 and 2.3. As cycling progresses, the GC profile transformed into a more gradual, “sloping” shape, suggesting increased amorphization of the active material.

To better visualize individual electrochemical processes, CV measurements were conducted with a voltage sweep rate of 0.1 mV s^–1^ within the same voltage window (Figure 2c). The peaks observed in the CV were assigned similar to previous studies.^9,10^ During the first charge, three oxidation peaks marked as **O1** (first plateau during the first charge in GC), **O2** (second plateau during the first charge in GC), and a shoulder-like peak O* were observed. Based on the previous reports, **O1** can be attributed to the oxidation of Fe^2+^, while the broader **O2** peak likely corresponds to the simultaneous oxidation of both the Fe^2+^ and Se^2–^ species. At higher potentials, the appearance of the shoulder O* is most likely associated with further oxidation of Se^2–^ to Se^–^ or Se^0^.^10,18^ During discharge, three reduction peaks labeled as R*, **R1**, and **R2** were observed. The shoulder-like peak R* and peak **R1** appearing around 2 V were assigned to the reduction of Fe^3+^ to Fe^2+^ and reduction of Se^0^ or Se^–^ to Se^2–^.^10^ Assuming that Fe is the only element responsible for the redox activity at low potentials, the **R2** peak could be possibly assigned to the reduction of Fe^2+^ to Fe^0^.

Starting from the second cycle, a new oxidation peak **O3** appears, which was previously assigned to the oxidation of elemental Fe.^18^ While the **O1** and **O2** processes continue to be present during subsequent cycles, the O* transition disappears. Similarly, during the second discharge, the R* peak disappears, and therefore, these peaks are related to structural changes occurring during the initial cycles. From the third cycle and onward, the CV profiles exhibit characteristics more common for amorphous materials, with a broad and less-defined peak spanning the original **O1** and **O2** regions (also shown in Figure S3 in the SI), resembling the behavior of Li_2_FeSO.^19^

Overall, the two-stage redox process observed during the first charge and discharge is likely linked to the electrochemical activity of both Fe^2+^ and Se^2–^, and thus, advanced structural characterization techniques are required to gain a deeper understanding of the structural evolution and redox mechanism of Li_2_FeSeO.

### 2.2 XRD and PDF Characterizations

#### 2.2.1 *Operando* XRD and PDF

The XRD and PDF analyses of Li_2_FeSeO provide important insights into its crystallinity, local coordination, and structural ordering upon cycling. *Operando* XRD and PDF measurements were performed using a specially designed *operando* cell.^27^ The cell was cycled at a current rate of 15 mA g^–1^ between 1 and 3 V *vs* Li/Li^+^. This current density was selected to obtain a sufficient amount of *operando* scans, and the results are illustrated in Figure 3a–f.

**Figure 3:**
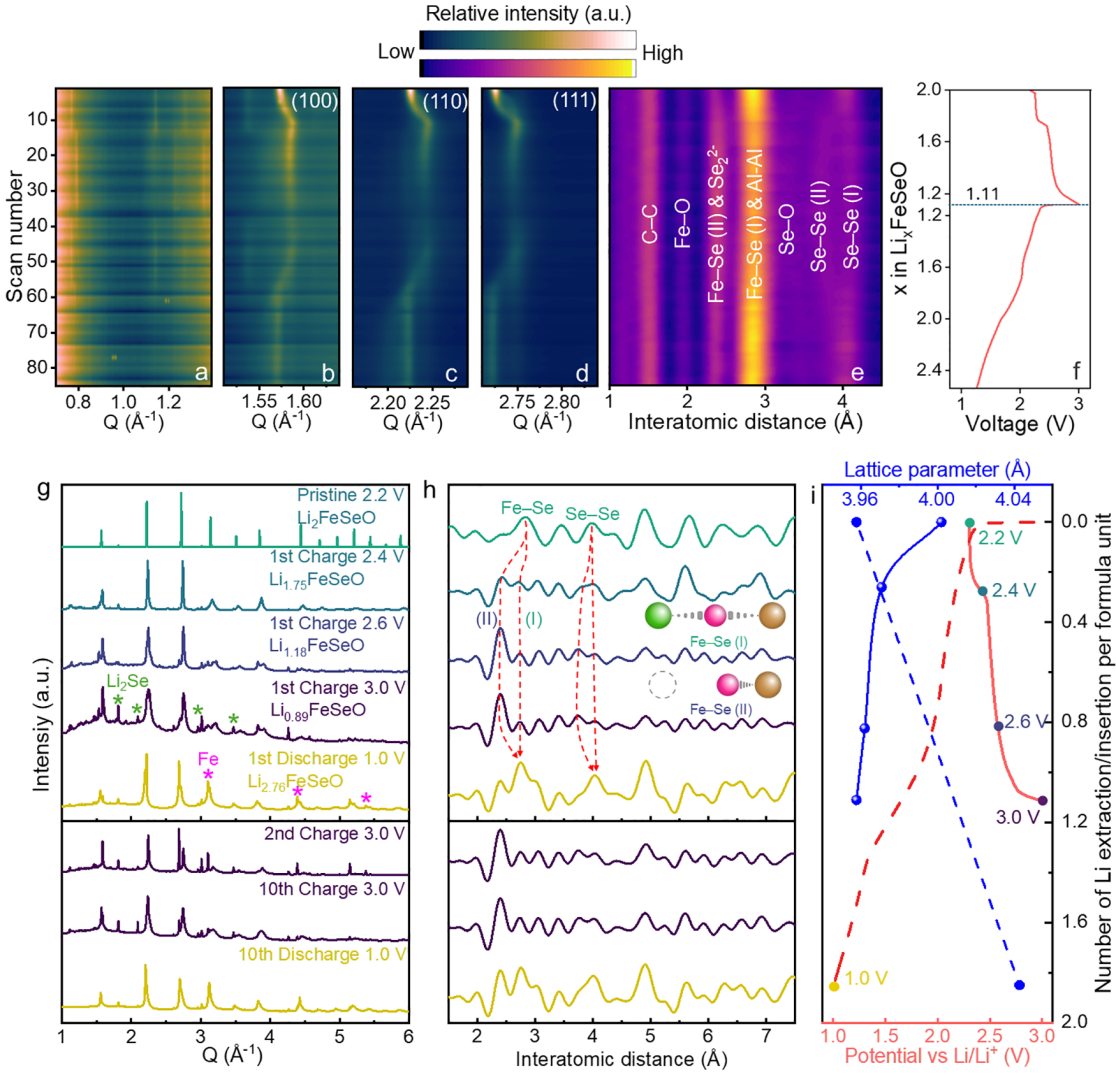
*Operando* and *ex situ* XRD and PDF. (a–d): *Operando* XRD at different *Q* regions; (e) *operando* PDF; (f) corresponding GC profile; (g) *ex situ* XRD at different states of charge/discharge, the asterisk symbols mark Li_2_Se and the possible formation of metallic Fe; (h) *ex situ* PDF in corresponding states; (i) electrochemistry of a coin cell used for *ex situ* measurements and comparisons of the lattice parameters in different states.

At first, the structural evolution of Li_2_FeSeO during the first cycle was examined by *operando* XRD (Figure 3a–d, where the diffraction pattern is separated into multiple panels for clarity). Figure 3a displays the low-*Q* region, where the appearance of weak diffraction peaks suggests the formation of possible superstructures due to ordered vacancies at the Li^+^ site, similar to Li_2_FeSO.^19^ The evolution of the main diffraction peaks (100), (110), and (111) is presented in Figure 3b–d, respectively.

During the first plateau of charging (2.2–2.4 V), the intensities of the main diffraction peaks decrease and shift toward higher *Q*, indicating a reduction in both volume and crystallinity. The absence of peak splitting suggests that the cubic phase remains intact throughout this stage of charge and further indicates the cation-disordered nature that leads to isotropic Li^+^ diffusion. Continuous peak sliding indicates a solid solution reaction at this stage. During the second plateau of charging (2.4–3.0 V), the intensities of the peaks continue to decrease and broaden, while the peak positions stay the same, making the material inhomogeneous through amorphization . Upon discharge, crystallinity was partially recovered, indicating that the amorphization process is not fully reversible to the pristine state. Starting from the second cycle, the XRD background raised, decreasing data quality and making the interpretation challenging (Figure S12 in the SI).

Due to the formation of amorphous phases, *operando* PDF analysis was conducted to complement the XRD analysis, which allowed one to focus on a detailed view of interatomic distance evolution during the first cycle as shown in Figure 3e. The major peaks corresponding to the interatomic distances are marked accordingly. Several peaks in the initial state can be related to Li_2_FeSeO. To distinguish between the pristine and delithiated structures, the original interatomic distances are labeled as (I), and the new distances emerging during delithiation are referred to as (II). Two main peaks corresponding to Fe–Se (I) interaction at 2.8 Å and Se–Se (I) interaction at 4.0 Å are marked, with the behavior of both peaks changing upon cycling. Upon delithiation, the intensity of the Se–Se distance decreased, reflecting distortion at Se sites. The Se–Se (I) peak splits into two peaks after the first plateau (2.4 V), where Se–Se (I) remains, while a split peak shifts and decreases to 3.75 Å in the fully charged state (3.0 V), which is marked as Se–Se (II). The peak at 2.41 Å is assigned to the shortened Fe–Se distance and marked as Fe–Se (II). The Fe–Se (II) peak intensity increases upon charging, while the intensity of the Fe–Se (I) peak decreases. The presence of Fe–Se (II) at the beginning of cycling likely stemmed from structural distortion caused by the slurry process. Further illustration of Fe–Se interaction upon cycling will be discussed in the following sections.

#### 2.2.2 *Ex Situ* XRD and PDF

While *operando* analysis offers a broad view of the structural evolution during cycling, the elevated background significantly limits the depth of analysis. To validate the *operando* data and to enable more detailed structural characterization, *ex situ* measurements were carried out. Coin cells were cycled at 50 mA g^–1^ and stopped at specific states of charge and discharge, and the potential steps were selected based on the redox peaks observed in CV: during the first charge—pristine (open circuit voltage ∼2.2 V), 2.4 V (corresponding to **O1** in CV), 2.6 V (**O2**), and 3.0 V (end of the first charge)—and during the first discharge—1.5 V (after **R1** where the discharge capacity is close to the charge capacity) and 1.0 V (end of discharge).

Figure 3g shows the XRD patterns of Li_*x*_FeSeO at various states of charge and discharge, where the value of *x* is determined based on the capacity of one of the coin cells used for making the *ex situ* capillaries. The corresponding GC curve is shown in Figure 3i, with the red solid line representing charge and the dashed line representing discharge. The XRD analysis conducted at different stages of delithiation aligns closely with the simulated XRD patterns for each Li^+^ concentration (Figure S17 in the SI). Furthermore, this comparison indicates that the delithiated Li_*x*_FeSeO structure preserves the integrity of the AP framework during the delithiation process.

During the first charge, the primary diffraction peaks broadened when ∼0.25 Li^+^ per formula unit were removed (corresponding to voltage rise to 2.4 V), and this broadening continued until cutoff voltage (3.0 V) was achieved. This was accompanied by a raised background, indicating the formation of amorphous species, which is consistent with the observations from *operando* XRD. In addition, within this set of samples, the peaks corresponding to Li_2_Se were observed and marked as green asterisks in Figure 3g. These peaks originate from original impurity in the material and are almost invisible in the XRD corresponding to the original electrode; however, as crystallinity disappears, these peaks become visible. Discharge down to 1.0 V allowed the excess Li^+^ intercalation up to the formation of Li_(2+0.76)_FeSeO. The crystalline structure was partially restored, as evidenced by the reversal of the characteristic AP peaks; in addition, this introduction of extra Li^+^ led to peak broadening. At a low voltage (<1.5 V), excess Li^+^ intercalation may lead to the formation of metallic Fe, and the corresponding Fe peaks are marked with pink asterisks. The formation of metallic Fe was also reported in other works.^18,28^ A zoomed-in Fe peak indexing is also provided in Figure S8 in the SI. During the second charge, stronger structural distortions with peak splitting of all major reflections were observed. The crystallinity continued to degrade with cycling, showing increased peak asymmetry up to 10th cycle. This suggests progressive and irreversible amorphization, as also indicated by the broadened peak profile in CV recorded during the third cycle (Figure 2c).

The change in the lattice parameter, indicative of the unit cell volume of crystalline Li_*x*_FeSeO during cycling, is shown in Figure 3i in blue lines, where the solid line corresponds to charge and the dashed line corresponds to discharge. The volumes were calculated from the (100) peak position, as performing a full refinement proved challenging due to significant amorphization following (de)­lithiation. During charging, the most substantial volume change occurs in the first plateau (2.2–2.4 V), where approximately 0.25 Li^+^ is extracted per formula unit. The lattice parameter slightly decreases from 4.00 to 3.97 Å. In the subsequent delithiation during the second plateau (2.4–3.0 V), the volume change slowed with the lattice parameter reducing from 3.97 Å to 3.96 Å as additional ∼0.87 Li^+^ is extracted per formula unit. During discharging, the lattice parameter expands to 4.04 Å down to 1.0 V with an additional ∼0.76 Li^+^ inserted per formula unit.

The *ex situ* PDFs in different states of charge/discharge are shown in Figure 3h. In the PDF measurements, significant changes were observed in the peaks, while the material transitioned from the pristine structure to 2.4 V charged state, where several new peaks emerged, indicating a substantial change of the local coordination environment. Among these, the prominent peak around 2.4 Å corresponding to the Fe–Se (II) interaction appeared after the first plateau and dominated the PDF pattern. The intensity of this peak decreased upon discharge, and the original peaks associated with the cubic AP phase reappeared while in a modified form. Subsequent cycles showed an irreversible formation of a peak at 2.41 Å in the discharged state, suggesting a new interaction other than Fe–Se (II), and possibly related to the Se–Se bond considering the strong intensity. Other notable peaks upon charging within the unit cell range include shortened Fe–Se (I) at 2.70 Å, shortened Se–O at 3.41 Å, and Se–Se (II) at 3.75 Å. This suggests the coexistence of the new phase alongside the distorted original AP phase. A new peak located at 3.07 Å, remained uncertain but is likely associated with Fe–Fe or Fe–Se interactions in the new phase following Fe and Se reorganization during delithiation. Its behavior is similar to that of the Fe–Se (II) peak, with its intensity increasing over subsequent cycles. The key changes from Fe–Se (I) to Fe–Se (II) during de/lithiation are illustrated in the inset of Figure 3h. During discharge down to 1.0 V, the cubic AP phase is partially restored with a distortion that changes the interatomic distances. It should be mentioned that the peak corresponding to Fe–Fe of metallic Fe (around 2,48 Å) was not observed, and this is possibly due to peak overlapping when extracting PDF data from XRD and is further discussed in Section S3 (in the SI). The observed consistency between *ex situ* and *operando* diffraction results indicates that structural relaxation has a minimal impact on the material’s behavior.

### 2.3 XAS Characterizations

#### 2.3.1 *Operando* XAS

While XRD and PDF provide information about long- and short-range atomic correlations, XAS offers additional elemental-specific insights about coordination, electronic configuration, oxidation state, and local symmetry of active materials upon cycling.^29^ XANES and FT-EXAFS data in the *operando* modality of Se and Fe *K*-edges during the first cycle are presented in Figure 4a–d.

**Figure 4:**
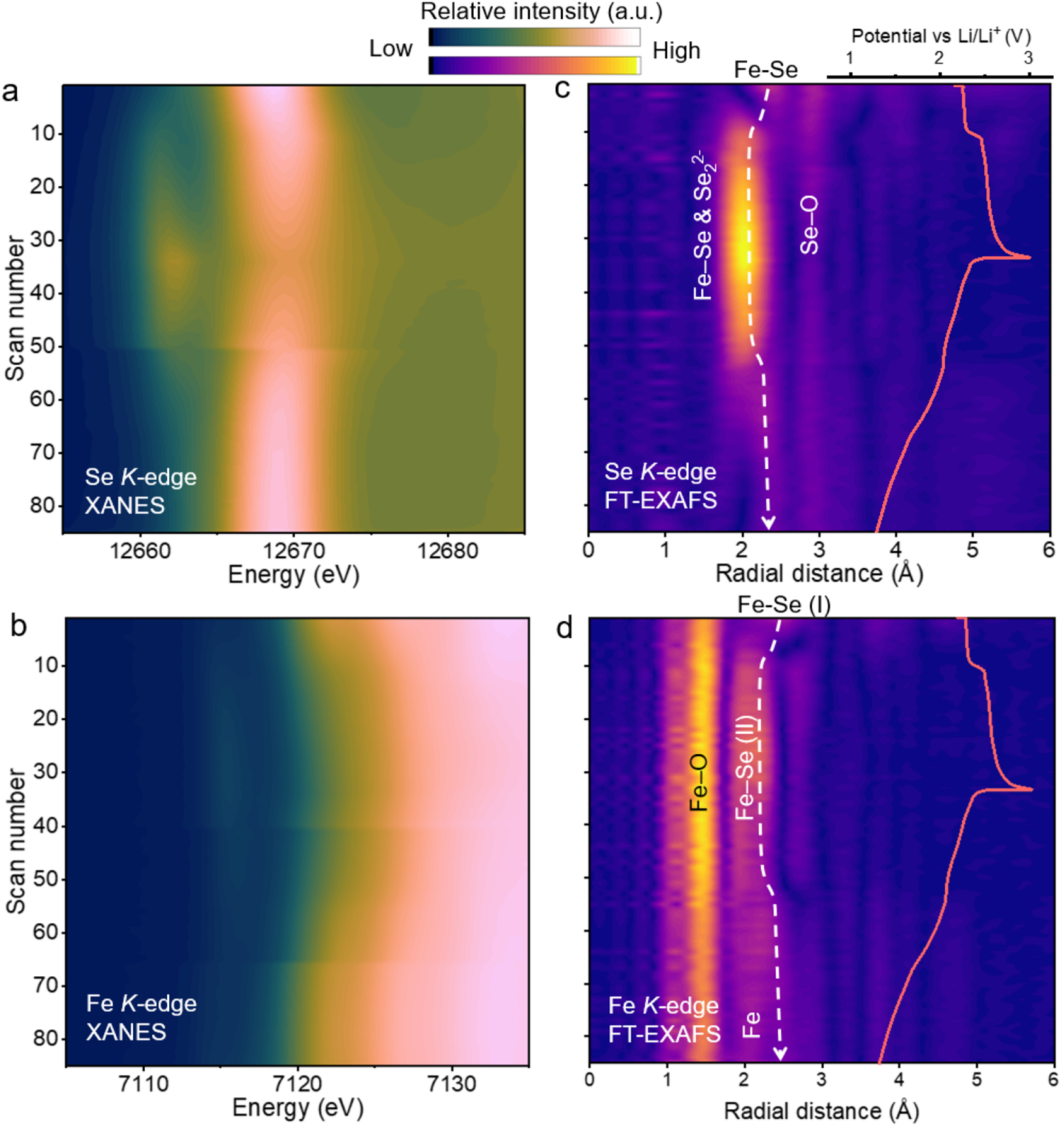
*Operando* XANES spectra of (a) Se and (b) Fe *K*-edges. *Operando* FT-EXAFS spectra of (c) Se and (d) Fe *K*-edges. The red lines are the corresponding GC profile that is the same as that shown in Figure 3f.

Figure 4a shows the *operando* XANES spectrum of Se *K*-edge. When measured during the first voltage plateau (2.2–2.4 V) of charging process, the weak shoulder intensifies into a pre-edge feature, while the edge peak shifts to higher energy. During the second voltage plateau (2.4–3.0 V), the pre-edge intensity continues to increase and shifts to higher energy, whereas the edge peak intensity drops sharply without significant change in the position. The emergence of the rising pre-edge feature, the shift in the edge position, and the decrease in the edge peak intensity indicate a progressive oxidation of Se^2–^ at the beginning of charging. This behavior partially resembles the observation in Li–Se batteries, where fully delithiated Se (Se^0^) displays a strong pre-edge feature that surpasses the intensity of the white line.^30^ In contrast, the pre-edge feature corresponding to the delithiated Li_2–*x*_FeSeO remains less intense than the white line. This observation suggests that the average oxidation state of Se in the delithiated state is lower than Se^0^, possibly closer to Se^–^. The behavior of Se at high voltage will be further discussed in the following sections. During discharge, the pre-edge and edge profiles reversed, though XANES for the lithiated state (around 1.25 V) showed reduced edge-peak intensity compared to one measured at the beginning of charging. The abrupt shifts in the peak position and intensity during discharge (scan >51) were likely due to energy calibration drift or sample displacement and do not represent intrinsic behavior of the material.

Figure 4b shows the *operando* XANES spectrum of the Fe *K*-edge. When measured during the first voltage plateau (2.2–2.4 V) in the charging process, the rising pre-edge for Fe *K*-edge indicates a structural distortion, where the edge profile changed significantly, indicating a strong local distortion. Both pre-edge and edge features indicated a coordination change and decreased centro-symmetry around Fe upon delithiation. The Fe pre-edge continued to intensify alongside an edge shift during the second voltage plateau (2.4–3.0 V), indicating the involvement of both Fe and Se in the redox process at high potentials. The Fe *K*-edge peak position stabilized around 2.5 V, suggesting that beyond this voltage Fe oxidation slows down, while Se oxidation starts to play a more significant role. Similar to Se, Fe is reduced upon discharging as the *K*-edge moved back to the low-energy region and the pre-edge feature decreased. *Operando* XANES of both Se and Fe *K*-edges, presented in a one-dimensional (1D) format, are shown in Figure S9, and a comparison of edge behaviors of Se and Fe is presented in Figure S10 (in the SI).

The Fourier transformed extended X-ray absorption fine structure (FT-EXAFS) data extracted from the *operando* XAS data set for both Fe and Se *K*-edges were used to analyze local structural changes during the first cycle, as shown in Figure 4c,d, respectively. The *k*-range used was between 3 and 11 Å^–1^ for Se and between 2.5 and 12 Å^–1^ for Fe. Note that due to phase shifts, peak positions in FT-EXAFS do not directly represent actual interatomic distances. Therefore, the interatomic distances obtained through FT-EXAFS require corrections of 0.3–0.5 Å in an extended fitting process to provide a more realistic real space coordination environment.^31–33^ Interaction between Se and Fe is subjected to substantial changes upon cycling, and the peaks that correspond to these major interactions are marked. During the first voltage plateau (2.2–2.4 V) of the first charge, the reduced intensities of the Fe–Se (I) interaction in both Se and Fe FT-EXAFS suggest decreased crystallinity of the material. Later, in the charging process, a pronounced structural change occurred, indicated by the appearance of a strong peak at approximately 2 Å in both Se and Fe FT-EXAFS corresponding to a shortened Fe–Se distance. Considering the phase shift, this peak has a similar behavior and interatomic distance as Fe–Se (II) observed in PDF, further confirming the shortened Fe–Se distance upon delithiation. The intensity of the peak corresponding to Fe–Se (II) continued to increase through the first charging process, indicating an appearance of a strong local ordering. Considering the peak raised with the increased pre-edge feature of Se *K*-edge, Fe–Se (II) may originate from the increased covalency between Fe and Se that relates to the hybridization of Fe 3d and Se 4p orbitals.^34^ During discharge, the intensity of this peak steadily decreased from 2.5 to 2.0 V, which corresponds to the reduction shoulder R* and peak **R1** in CV. This was followed by the reversible emergence of the peak associated with Fe–Se (I). However, the original cubic AP structure was not fully restored, as shown by the less-defined peaks at the end of discharge, indicating irreversible structural distortion after the first cycle. In addition to Fe–Se interaction, changes in Fe–O distances such as shortening during charge and elongating during discharge were also observed. Upon further discharge down below 1.9 V, where ∼1 Li^+^ per formula unit was reinserted, a broad new peak appeared around 2.1 Å, this peak may correspond to the formation of metallic Fe. The detailed illustration of low-voltage behavior will be further discussed in the *ex situ* FT-EXAFS parts in Sections 2.3.2 and S4. *Operando* XAS corresponding to the second cycle exhibiting similar behavior as the first cycle is presented in Figure S11 in the SI.

*Operando* XAS and XRD at both Fe and Se *K*-edges revealed a reversible dual-redox process involving both Fe and Se as well as structural distortion during the first cycle, which was accompanied by a pronounced change in the Fe–Se interatomic distance. During discharge, both Se and Fe were reduced, leading to an elongation of the Fe–Se distance and the reappearance of the original cubic AP phase. However, the potential instrument-induced edge shift in *operando* XANES hinders further interpretation, emphasizing the need for *ex situ* measurements.

#### 2.3.2 *Ex Situ* XAS

While *operando* measurements provide valuable insights, the strong background from the *operando* cell components and modifications required for X-ray studies can impact the electrochemical performance. This is evident in the abnormally high capacity observed during the first discharge of the *operando* cell (Figure 3f), the sudden rise in the *operando* XRD intensity in the low-potential region (Figure S12 in the SI), and the edge shift in *operando* XANES, which highlighted the importance of complementary *ex situ* measurements to better characterize the redox process and structural evolution in the conventional coin cells.

Figure 5a illustrates the Se *K-*edge XANES during charge (top) and discharge (bottom) for the first cycle. Upon delithiation, the coordination environment around Se^2–^ rapidly changes, inducing significant local distortion. The pre-edge region is sensitive to local structural change and therefore provides information about local coordination of Se^2–^. The appearance of the pre-edge feature (marked as peak ***a***) indicates transformation of Se^2–^ surroundings from central-symmetric to noncentral-symmetric. This pre-edge corresponds to the hybridization of Se 1s→4p and Fe 1s→3d transitions, as shown in the inset in Figure 5a.^16^ Comparing the samples extracted at 2.4, 2.6, and 3.0 V, the intensities of both the pre-edge and edge peak (marked as **b**) vary significantly, indicating the oxidation of Se^2–^. Thus, the oxidation of Se^2–^ begins at the initial stage of charging and plays a crucial role in the redox processes. The contribution of Se is less prominent during the first voltage plateau (2.2–2.25 V) but becomes more significant in the second voltage plateau (2.4–3.0 V). This is evident from the pronounced changes in the pre-edge feature, which corresponds to the oxidation peak **O2** and the shoulder O* observed in CV. Upon discharge, the pre-edge intensity decreases along with the increased edge peak intensity, indicating a reversible redox process for Se^2–^ during the first cycle.

**Figure 5:**
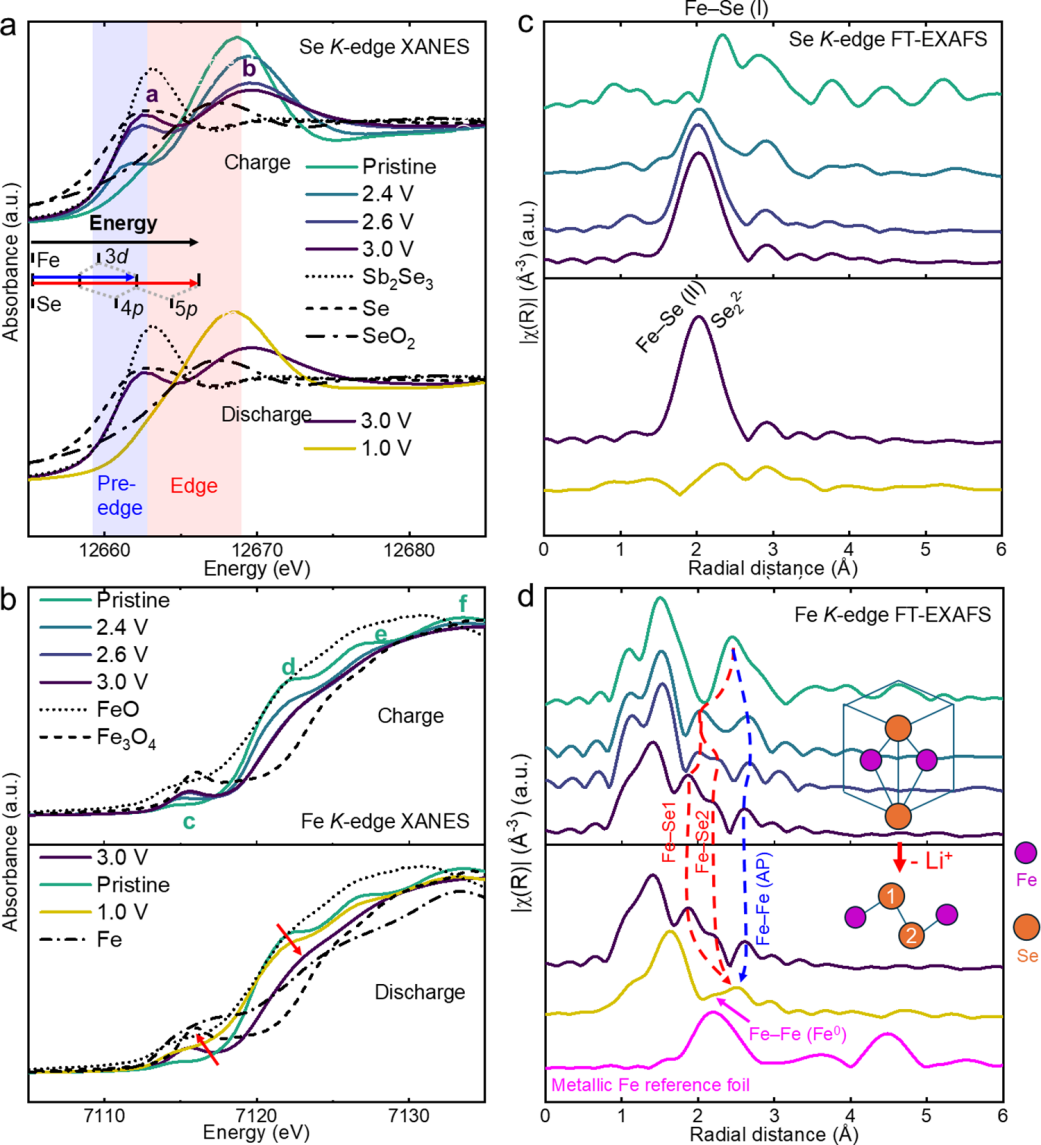
*Ex situ* XAS. (a, b) *Ex situ* XANES spectra of Se and Fe *K*-edges during the first cycle; (c, d) corresponding FT-EXAFS results for the first cycle, respectively.

Figure 5b shows Fe *K*-edge XANES for the samples extracted during the first cycle. Upon charging, the pre-edge intensity increases and shifts to higher energy, and the edge profile changes as the multistage shoulders disappear upon charging to 2.4 V accompanied by a slight shift of the edge position. This shift of the pre-edge and the edge is indicative of the oxidation of Fe^2+^ and a strong change of local coordination around Fe^2+^ that corresponds to peak **O1** in CV. Beyond 2.4 V, both Fe and Se ions actively participate in the oxidation process as the edge positions shift to higher energy. The distinct edge movement of the Fe *K*-edge observed during the first and second voltage plateaus suggests two different oxidation processes. During the first voltage plateau, Fe^2+^ is partially oxidized to Fe^3+^ and undergoes migration and reordering, likely occupying vacant Li^+^ sites, while the second voltage plateau shows a steady oxidation process with a reduced site movement. This analysis of the gradual oxidation state is further supported by the calculated Bader charges, as shown in Figure S19 in the SI. Direct comparison of valence states of Fe with the reference samples is challenging due to differences in coordination environments; however, based on the edge shift, it is unlikely that all Fe^2+^ oxidized to Fe^3+^ upon delithiation, and the additional Se^2−^/Se^−^ redox activity contributes to the high capacity of the material. During discharge, Fe^3+^ and Se^–^ are reduced. Further lithiation down 1.0 V does not significantly change the Se *K*-edge feature as reduction of Se^2–^ is unlikely. For Fe *K*-edge, the edge revolves around 7121 V, as indicated by the arrows, and such behavior resembles the conversion reaction as observed in Fe_1–*x*_S,^35^ which suggests the formation of Fe^0^.

Se and Fe *K*-edge FT-EXAFS spectra are presented in Figure 5c,d, respectively, and the corresponding EXAFS spectra are shown in Figure S13 in the SI. The peak movements are consistent with the *operando* data. The shift of the Fe–Se peak position in Fe FT-EXAFS slightly differs from that in Se FT-EXAFS, where the strong peak corresponding to the Fe–Se (II) distance in Se FT-EXAFS remains unchanged beyond 2.4 V, while the same peak in Fe FT-EXAFS moves to a shorter distance when the volatge changed from 2.6 to 3.0 V. This suggests that the peak at 2 Å in Se FT-EXAFS upon delithiation arises from multiple origins and not only Fe–Se interactions, which will be further discussed in Section 2.4. Upon discharge to the 1.0 V state, a small peak is marked, which may correspond to the metallic Fe–Fe distance. It should be noted that the origin of this peak may also be partially attributed to the remaining of Fe–Se (II). The FT-EXAFS fittings were carried out to better demonstrate the structural evolution and are shown in Figures S15 (Se *K*-edge) and S16 (Fe *K*-edge) in the SI.

*Ex situ* XANES and FT-EXAFS data revealed significant changes in the oxidation state and local coordination of Se and Fe during the first cycle. XANES demonstrates a dual-redox mechanism involving both Fe^2+^ and Se^2–^. FT-EXAFS showed pronounced structural changes, particularly the decrease in the Fe–Se distance. The increased peak intensity in FT-EXAFS at the delithiated state suggests enhanced local structural ordering. The analysis of subsequent cycles (Section S3) revealed an irreversible change in the coordination environment of Se at the 10th cycle. In Se FT-EXAFS, the strong peak at 2 Å dominates the structure in both charged and discharged states; however, the peak in the discharged state has no corresponding peak in Fe FT-EXAFS. This suggests an interaction which is distinct from Fe–Se but with a similar interatomic distance, possibly the Se–Se bond. The strong agreement between the PDF and FT-EXAFS data further supports the reliability of the proposed structural evolution.

### 2.4 Structural Evolution

XRD, PDF and XAS analyses revealed the formation of a new, distorted phase after the first voltage plateau (2.2 - 2.4 V) during the first charge. Significant local reordering is evident from the decreased Fe–Se interatomic distances accompanied by reduced central symmetry for both Fe and Se with pronounced coordination changes. However, the strong local distortion associated with Fe–Se interaction does not compromise capacity retention, suggesting that this new phase plays a crucial role in material’s high capacity and cyclability.

As previously discussed, the strongest peak observed in PDF and FT-EXAFS for the delithiated state is not solely attributed to the Fe–Se interaction but may also indicate bonding between Se–Se as a result of Se^2–^ oxidizing to Se_2_^2–^. To verify this, Raman spectra of pristine and delithiated (3.0 V) samples were obtained and are presented in Figure 6a. The noticeable peak at 247 cm^–1^ is likely associated with a vibrational mode of the pristine Li_2_FeSeO. Upon delithiation, several peaks associated with perselenide Se_2_^2–^ were observed, as their intensities became more pronounced upon delithiation. The peaks at 175 cm^–1^ and 216.5 cm^–1^ correspond to Se–Se rotational and stretching modes, respectively.^36–40^ Additionally, a new Raman peak at 281.5 cm^–1^ likely results from the increased surface activity that occurs as the particle size decreases at higher potentials, leading to significant changes in surface interactions. As a result, a new local structure was formed with the rearrangement of Fe^3+^ and Se_2_^2–^.

**Figure 6:**
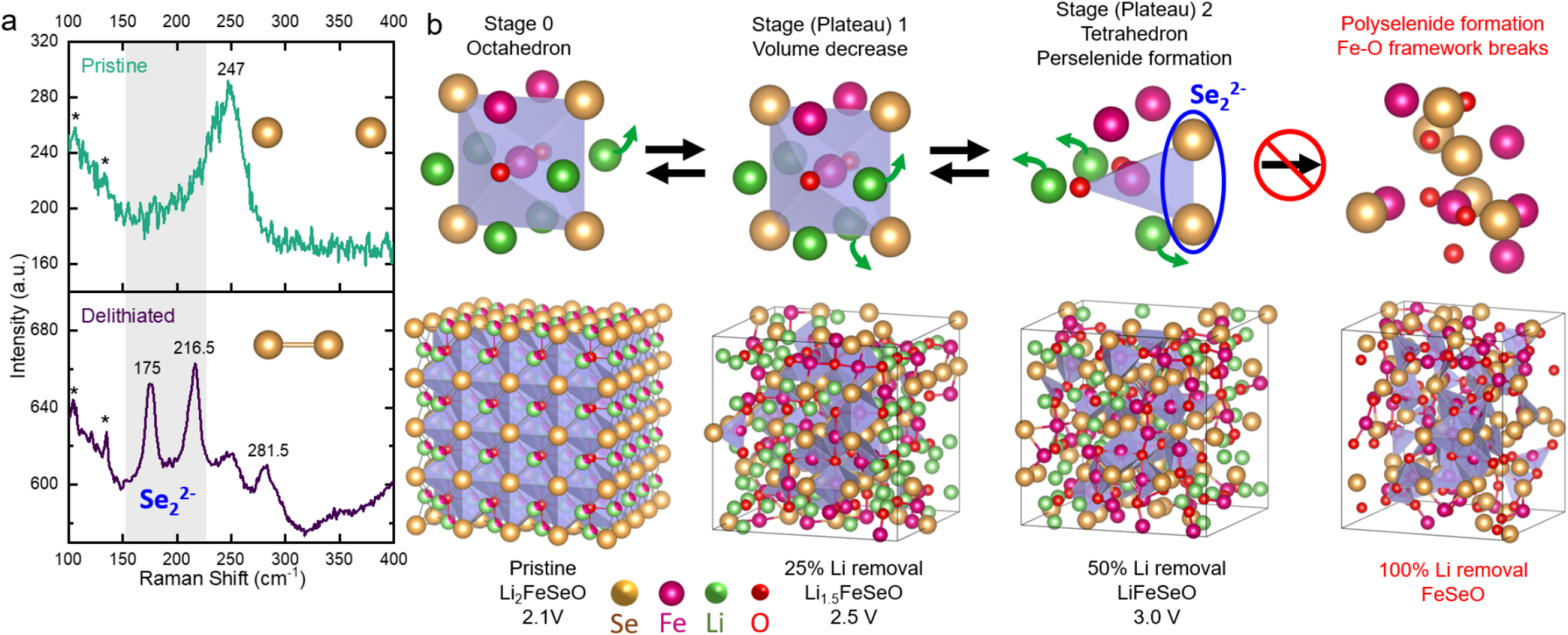
(a) Raman shift and (b) structural evolution of Li_2_FeSeO during the first charge.

To complement the characterization techniques and provide a better visualization of the (de)­lithiation processes in Li_*x*_FeSeO, a series of DFT calculations were performed. The lower panel of Figure 6b illustrates the structural changes during the first cycle of Li_*x*_FeSeO representing calculated structures in varying states of delithiation (*x* = 2.0, 1.5, 1.0, and 0.0). The formation of Se pairs (Se_2_^2–^) was observed at *x* = 1.0 confirming the results obtained through Raman spectroscopy. This observation further suggests that the strong peak in Se FT-EXAFS is partially attributed to Se–Se interactions and possibly the main contribution to the peak in the discharged state at the 10th cycle (Figure S14 in the SI). Given Fe’s consistent interaction with two oxygen atoms throughout the cycling and the proximity of Se to Fe, Fe was selected as the central coordination atom to present the delithiation process. As shown in the upper panel in Figure 6b, Fe adopts an octahedral coordination in pristine Li_2_FeSeO, forming a Fe–O_2_Se_4_ local configuration, where Fe–Se (I) bonds exist. During the first charging plateau (2.2–2.4 V), the Fe–O and Se frameworks undergo structural distortion, leading to reduced crystallinity and volume. Upon further delithiation (25–50% of Li^+^ removal), the second voltage plateau is reached. The loss of Li^+^ ions causes Se^2–^ to move closer to Fe, shortening the Fe–Se (I) distance and adapting a type of Fe–Se (II). This movement also displaces oxygen atoms, changing the linear −O–Fe–O– configuration into a zigzag chain (see also Figure S20 in the SI). Together, two adjacent Se^–^ ions and two O^2–^ ions surrounding Fe^3+^ create an Fe–Se_2_O_2_ tetragonal local motif. To compensate for the delithiation, the perselenide pair Se_2_^2–^ is formed within the tetrahedron.

Further hypothetical delithiation to 100% results in the formation of FeSeO clusters (polyselenides), which disrupt the crystallinity and transition the structure into a fully amorphous phase. This explains the inability to achieve the complete delithiation of AP cathodes. To validate the proposed local structural distortion, FT-EXAFS fittings for pristine and charged states of Li_*x*_FeSeO are presented and discussed in Section S4. The reversible formation of Fe–Se_2_O_2_ tetragonal motifs during cycling enables the reversible electrochemical process.

Importantly, the Fe–O framework remains intact as a structural host for Se upon delithiation, which prevents polyselenide formation. This mechanism suppresses degradation commonly observed in Li-chalcogenide batteries,^41^ potentially promoting AP as a strategy to prevent polysulfide/selenide migration in future development of Li–S and Li–Se batteries.

In summary, the delithiation and lithiation processes can thus be summarized as follows.

Delithiation:

1 $${\mathrm{Li}}_{2}\mathrm{FeSeO}\to {\mathrm{Li}}_{2-x}\mathrm{FeSe}+x{\mathrm{Li}}^{+}+xe^{-}\left(<3.0V,x<1.3\right)$$

Lithiation:

2 $${\mathrm{Li}}_{2-x}\mathrm{FeSeO}+x{\mathrm{Li}}^{+}+xe^{-}\to {\mathrm{Li}}_{2}\mathrm{FeSeO}\left(>1.5V\right)$$

$${\mathrm{Li}}_{2}\mathrm{FeSeO}+y{\mathrm{Li}}^{+}+ye^{-}\to {\mathrm{Li}}_{2+y}{\mathrm{Fe}}_{1-\frac{1}{2}y}\mathrm{SeO}+\frac{1}{2}y\mathrm{Fe}\left(<1.5V,y<0.8\right)$$ 3

Several redox peaks observed in CV can be interpreted based on the proposed redox process and structural changes during the first and second cycles. During the first charge, **O1** corresponds to the oxidation and coordination change of Fe^2+^ and Se^2–^, with a preference for Fe^2+^ oxidation, accompanied by a volume decrease and structural distortion, and **O2** and O* correspond to the combined oxidation of both Se^2–^ and Fe^2+^. Based on the fact that Se_2_^2–^ is dominating the structure at 3.0 V, where 1.2 Li has been extracted per formula unit, one would expect that oxidation of Se^2–^ to Se_2_^2–^ is a key contribution to the high capacity. **O2** is also associated with the formation of the Fe–Se_2_O_2_ tetrahedral local motif. During discharge, R* and **R1** correspond to the reduction of Fe^3+^ and Se^–^, accompanied by the reverse of the tetrahedral Fe–Se_2_O_2_ to original Fe–O_2_Se_4_ octahedral configuration, and **R2** marks the conversion process that forms metallic Fe as redox pairs of Fe^2+^/Fe^0^ together with **O3**. The merging of **O1**, **O2**, and O* peaks in subsequent cycles suggests an irreversible structural distortion. This distortion leads to the increase of Fe–Se (II) and Se_2_^2–^ contents and a higher population of tetrahedral coordination within the structure.

## 3 Conclusions

In this work, we correlated the electrochemistry of AP Li_2_FeSeO with structural evolution, focusing on both crystallinity and local order using multiple characterization techniques. In the voltage range of 1–3 V, the analysis has shown that the cyclability of the high-capacity Li_2_FeSeO cathode is attributed to the dual-redox activity of both Se and Fe. Upon delithiation, Se plays a crucial role in the redox process at high voltage by transitioning between selenide (Se^2–^) and perselenide (Se_2_^2–^) states, and the amount of perselenide ions increases upon subsequent cycling; while Fe^2+^ is oxidized to Fe^3+^ in the low-voltage region. As a result of Li^+^ vacancies formed during the delithiation process, a notable local structural distortion occurs around the Fe site. Upon lithiation, the Se_2_^2–^ ions are reduced to Se^2–^ and Fe^3+^ to Fe^2+^, which further reduced to Fe^0^ in the low-potential range. Furthermore, the presence of the dual-redox couple facilitates robust Fe–O framework formation, which is identified as the main reason for structural stability during the electrochemical cycling. We believe that the comprehensive investigation highlights valuable insights into how Li_2_FeSeO operates, which may be applicable to other AP cathode materials, demonstrating their potential for future applications.

## 4 Methods

### 4.1 Synthesis

A one-step solid-state reaction was used to synthesize APs. Due to the air and moisture sensitivity of APs, precursor preparation was performed in an argon-filled glovebox (MBRAUN) with O_2_ and H_2_O levels maintained below 0.5 ppm. Li_2_O (97%, Sigma-Aldrich), Fe (99%, Sigma-Aldrich), and Se (99.5%, Sigma–Aldrich) were mixed in a ratio of 1.05:1:1, with a total weight of approximately 1 g, and ground in an agate mortar for 10 min. The resulting powder was loaded into an alumina crucible and placed inside a silica ampule. The ampule was initially sealed with a rubber stopper and evacuated using a 50 mL syringe. It was then removed from the glovebox and instantly sealed with a hydrogen torch within 5 min. The vacuum inside the ampule was confirmed using a spark tester.

The sealed ampule was heated in a furnace at a rate of 1.2 °C min^–1^ from room temperature to 750 °C, held at 750 °C for 10 h, and then allowed to naturally cool to room temperature. After cooling, the ampule was transferred back into the glovebox, where the powder was extracted and ground in an agate mortar for 10 min prior to characterization.

### 4.2 Coin Cell Preparation

Due to the air-sensitive nature of AP materials, the slurry preparation process must be conducted in an inert atmosphere. Grounded Li_2_FeSeO powder, poly­(vinylidene fluoride) (PVDF, Sigma-Aldrich), and Super P (TIMCAL) were mixed in an 8:1:1 weight ratio to a total amount of 200 mg. The mixture was dissolved in 1.8 mL *N*-methyl-2-pyrrolidone (NMP, Sigma-Aldrich). Manual mixing resulted in low-quality slurry with visible chunks and bubbles, making it difficult to coat and leading to poor electrochemical performance. To improve the slurry quality and ensure homogeneous distribution, two methods were developed:

1. Mixing using a magnetic stirrer: performing inside a glovebox under air- and moisture-free conditions. The process is over 100 h but produces high-quality slurry.
2. Using a planetary ball mill: quicker but involves strong kinetic energy that possibly induces structural distortion.

Further details on the slurry preparation are provided in Section S1. Due to the large quantity required for *ex situ* synchrotron measurements , Li_2_FeSeO for these measurements was prepared using a ball mill, while slurries for *operando* measurements and electrochemical tests were mixed inside the glovebox. After drying, the active mass loading per electrode ranged from 1.2 to 2.3 mg cm^–2^.

Electrochemical performance was evaluated in a half-cell configuration using lithium foil (China Energy Li Co., with purity: ≥99.9%, diameter: 15.5 mm, and thickness: 0.4 mm) as the counter electrode. CR2032 coin cell components (stainless steel 304, Neware) and glass microfiber separators (16 mm, Whatman) were utilized. The separators were soaked in 80 μL of electrolyte. Galvanostatic cycling (GC) was performed using a Neware CT-4008T-5 V10 mA-164 battery tester within a voltage range of 1.0–3.0 V *vs* Li/Li^+^. The initial voltages of the coin cells were in the range of 2.05–2.21 V. A 5 h rest period was applied before cycling for all coin cells. CV was conducted with a Biologic MPG2 battery cycler over the same voltage range at a sweep rate of 0.1 mV s^–1^. A gradual structural distortion of Li_2_FeSeO was observed after the slurry process and cell assembly, which is possibly related to Li leaching, and is discussed in Sections S2 and S3.

### 4.3 *Operando* Characterizations

A laboratory manufactured cell with glassy carbon windows was used for *operando* measurements based on the design from ref 27. A 20 mm separator (Whatman) with 160 μL electrolyte (LiPF_6_ in EC:EMC in a 3:7 vol % with 2 wt % VC and 10 wt % FEC) and Li metal foil were used for *operando* cells. GC measurements were performed using an SP150 battery cycler (Biologic).

#### 4.3.1 In-House *Operando* Measurements

A Bruker D8 A25 powder diffractometer (Mo source; λ_Kα1_ = 0.7093 Å; λ_Kα2_ = 0.7136) was used in transmission mode. XRD was measured by continuous scans (5 min scan^–1^, *Q* range between 1.12 and 3.48 Å^–1^). The cell was cycled at 10 mA g^–1^ in a voltage range of 1–3 V.

#### 4.3.2 Synchrotron *Operando* Measurements

Li_2_FeSeO electrodes were prepared using the method described in the Coin Cell Preparation section. The electrodes were triple-packed inside coffee bags sealed with a heat sealer. Synchrotron measurements were carried out at beamline BM31 ^42^ of the European Synchrotron Radiation Facility (ESRF) in Grenoble, France, where a combined X-ray total scattering (XRD and PDF) and XAS allows for studying real-time structural evolution of battery materials during cycling.^43^ For X-ray total scattering, a Dectris Pilatus 3X 2 M CdTe detector and monochromatic radiation with a wavelength of 0.25995 Å were used; the Two-dimensional (2D) diffraction data were integrated with PyFai and converted to PDFs with PDFgetX3. The distances between the sample and detector were 0.843 m for XRD and 0.273 m for PDF, respectively. For pattern fitting of the XRD and PDF data, Topas version 7 was used.For XAS, a subset of 3 out of 16 prefilled ionization chambers were used. The energy threshold ranged from 12.53 to 13.31 keV for the Se *K*-edge and from 7.01 to 7.76 keV for the Fe *K*-edge, with step sizes of 0.7 and 0.5 eV, respectively. XRD, PDF, and XAS were conducted in transmission mode. Each capillary scan set consisted of one XRD scan (30 s per scan), ten PDF scans (30 s per scan), and two XAS scans (162 s per scan) on both Fe and Se *K*-edges, respectively. Ten PDF scans were averaged, and two XAS scans were normalized and merged to increase the data quality. The total time required to complete one scan set was approximately 18 min. To increase the XRD and XAS intensities, an electrode with active mass loading of 7 mg was used. Athena and Artemis software were used for XAS data plotting and fitting.^44^ The edge energy was chosen as the maximum of the first derivative of μ­(E) for FT-EXAFS plotting.

The empty *operando* cell with glassy carbon windows was used for background removal in *operando* PDF measurements.

### 4.4 *Ex Situ* Characterizations

#### 4.4.1 In-House XRD

XRD (Bruker D8, Cu source, λ = 1.54 Å) was used for *ex situ* structure characterizations of Li_2_FeSeO. Data were collected over the range of 10–90° (2θ) using Cu Kα radiation in a fluorescence mode. To prevent oxidation, the sample holder was covered with a plastic dome before transferring out from the glovebox.

#### 4.4.2 SEM

SEM (Hitachi SU8230) was used for surface morphology and size distribution study; all sample preparations were carried out inside the glovebox and loaded into the SEM chamber immediately, and the total air-exposure time was under 2 min. The voltage used in SEM was between 2 and 3 kV, and the working distance was 10 mm.

#### 4.4.3 Synchrotron *Ex Situ* Characterizations

Li_2_FeSeO in 1 mm borosilicate glass capillaries (Hilgenberg) were measured. Coin cells with Li_2_FeSeO cathodes were cycled at 50 mA g^–1^ and stopped at several characteristic states of charge/discharge, the cells were then disassembled, the powder was scraped off from the current collectors and packed in the capillaries together with reference samples inside Ar-filled glovebox. The capillaries were loaded on a vertically aligned capillary rack for measurments. An Ar-filled empty capillary was used for background removal in *ex situ* PDF measurements. Each scan set used the same measurement route as described in the *Operando* Measurements section.

#### 4.4.4 Raman Spectroscopy

The main purpose with the Raman spectroscopy measurements was to verify the possible formation of perselenide Se_2_^2–^. Powders from pristine and delithiated (at 3.0 V) electrodes were scraped off, placed on a special glass slide (BK7) with a spherical groove, and enclosed in an argon atmosphere by a 0.15 mm thin rectangular cover glass (BK7) sealed with Apiezon L grease.

A helium–neon laser (632.8 nm) filtered *via* a 1200 rules mm^–1^ grating served as an excitation light source for the measurement. Raman measurements were undertaken using a Horiba Jobin–Yvon T64000 spectrograph working in single mode using a 900 rules mm^–1^ grating in a fixed position. An entrance slit width of 100 μm combined with a liquid nitrogen-cooled Symphony II CCD detector with 2.56 μm pixel size and 64 cm focal length led to a spectral width of 3.6 cm^–1^. The laser power at the sample was measured to be 1 mW, and an Olympus 50× objective was used. For the delithiated sample, a 7 × 7 square grid of points separated by 3 μm distance was mapped. 49 individual spectra were subsequently cleaned for spikes, averaged, and scale-corrected against a reference spectrum of 4-acetamidophenol (paracetamol). Here, each spectrum was an average of 10 exposures of 120 s. The pristine spectrum was an average of two sample points, each measured with 5 times averaging 120 s of exposure.

#### 4.4.5 Computational Details

We carried out *ab initio* calculations using the Vienna Ab initio Simulation Package (VASP),^45^ with the disordered occupancy of Li and Fe in Li_2_FeSeO, which was modeled using the Supercell program.^46^ For all computations, the Perdew–Burke–Ernzerhof (PBE) generalized gradient approximation (GGA) was employed as the electronic exchange-correlation functional.^47^ An energy cutoff of 520 eV was established for the plane-wave basis expansion. During the structural optimization process, a *k*-point mesh of 4 × 4 × 4 was utilized, following the Monkhorst–Pack grid method. The maximum force tolerance was set at 0.01 eV Å^–1^, and the energy difference criterion for electronic-state convergence was 1.0 × 10^–7^ eV atom^–1^.

## Supplementary Material



## Data Availability

The data for this publication, including electrochemistry, X-ray characterizations, Raman spectra, and DFT calculations, will be available at dataverse.no.^48^ Python scripts used for visualization of electrochemical performance and home-lab *operando* XRD measurement are available on GitHub.^49^ All raw files from the synchrotron experiments are stored at data.esrf.fr and will automatically be available in 2025–2027.^50^

## Notes

SEM characterizations; slurry process; electrochemical measurements; *ex situ* and *operando* XRD and XAS analyses; EXAFS fittings; and DFT calculations (PDF)
