# Supplementary material for: Role of Dual-Redox Couples in Antiperovskite Li2FeSeO Cathodes
Source: Chem Mater. 2025 Nov 11;37(22):9091–104. doi: 10.1021/acs.chemmater.5c01341 (PMC12659018; doi:10.1021/acs.chemmater.5c01341)
Supplement: Supplementary file 1 [file cm5c01341_si_001.pdf]

The Role of Dual Redox Couple in Antiperovskite Li_2FeSeO Cathodes

Tian Dai[1*], Heesoo Park[1], Anders Brennhagen[1], Niels Højmark Andersen[2], Marco Giorgetti[3], Martin Valldor[1], Alexey Y. Koposov[1*]

1. Centre for Material Science and Nanotechnology, Department of Chemistry, University of Oslo, P.O. Box 1033, Blindern, 0371, Oslo, Norway
2. Department of Chemistry, University of Oslo, P.O.Box 1033, Blindern, 0315, Oslo, Norway
3. Department of Industrial Chemistry, University of Bologna, Campus Navile, Via Piero Gobetti 85, Bologna 40139, Italy

Supporting information

Content

Section S1 Morphology and slurry preparation 3
Figure S1: SEM. 3
Figure S2: Illustration of two slurry making processes. 4
Section S2 Electrochemistry 6
Figure S3: Comparison of CV profiles for the first three cycles and onwards 6
Figure S4: Rate performance of Li_2FeSeO 7
Figure S5: Relaxation of Li_2FeSeO cathode in the coin cell 8
Figure S6: Capacity of the first charge and discharge 9
Section S3 X-ray characterizations 10
Figure S7: Tracking of structural change at different stages of cell assembly 10
Figure S8: Fe peak indexing 11
Figure S9: *Operando* XANES of Se and Fe *K*-edges. 12
Figure S10: Comparison of redox behavior of Fe and Se 13
Figure S11: *Operando* XAS and XRD of Li_2FeSeO during the 2nd cycle 15
Figure S12: Increased XRD intensity upon lithiation 16
Figure S13: EXAFS of *ex situ* measurements 17
Figure S14: *Ex situ* XAS of Li_2FeSeO at different cycles 19
Table 1: Interatomic distances 20
Section S4 Fittings and calculations 21

Figure S15: FT-EXAFS fitting of *ex situ* Se *K*-edge measurements 21
Figure S16: FT-EXAFS fitting of *ex situ* Fe *K*-edge measurements 22
Table 2: EXAFS fitting parameters 24
Figure S17: Simulated XRD. The structures calculated through DFT were used. 26
Figure S18: Simulated histogram 27
Figure S19: Simulated histogram of calculated Bader charge..28
Figure S20: Simulated Fe–O distances and angles upon delithiation.......................................29
References...30

Section S1 Morphology and slurry preparation

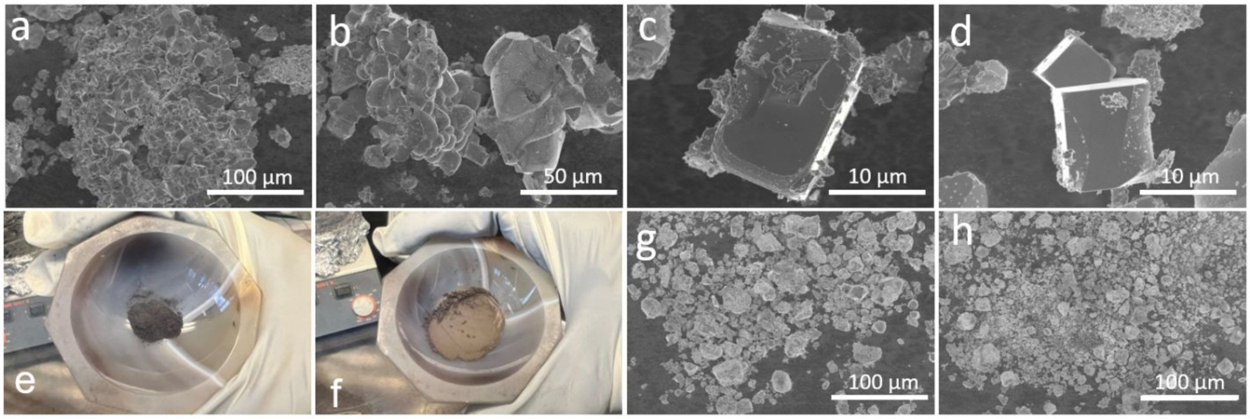

Figure S1: SEM. (a-d) SEM images of pristine Li_2FeSeO powder, (e, f): Color change before (left) and after (right) grinding. (g) SEM image of grounded powder; (f) SEM image of ball milled powder.

Figures S1a-1d show the morphology of Li_2FeSeO. The pristine powder tends to form chunks with sizes over 100 µm (Figures S1a and 1b), most of the single crystals found in the powder have the shapes of cubes or cuboids. Figures S1e and 1f show the powder morphology before and after manual grinding for 10 min, which is also accompanied by the color changes from black to dark brown, as reported previously.[1] The average particle size decreased to approximately 10 µm after grinding. To further reduce particle size, some samples underwent ball milling (using Pulverisette P23) at 20 Hz for 20 min and the corresponding SEM image is shown in Figure S1h. The ball milling was performed inside the glovebox. Approximately 500 mg of powder was loaded with 3 mm stainless steel balls at a sample-to-ball weight ratio of 1:10.

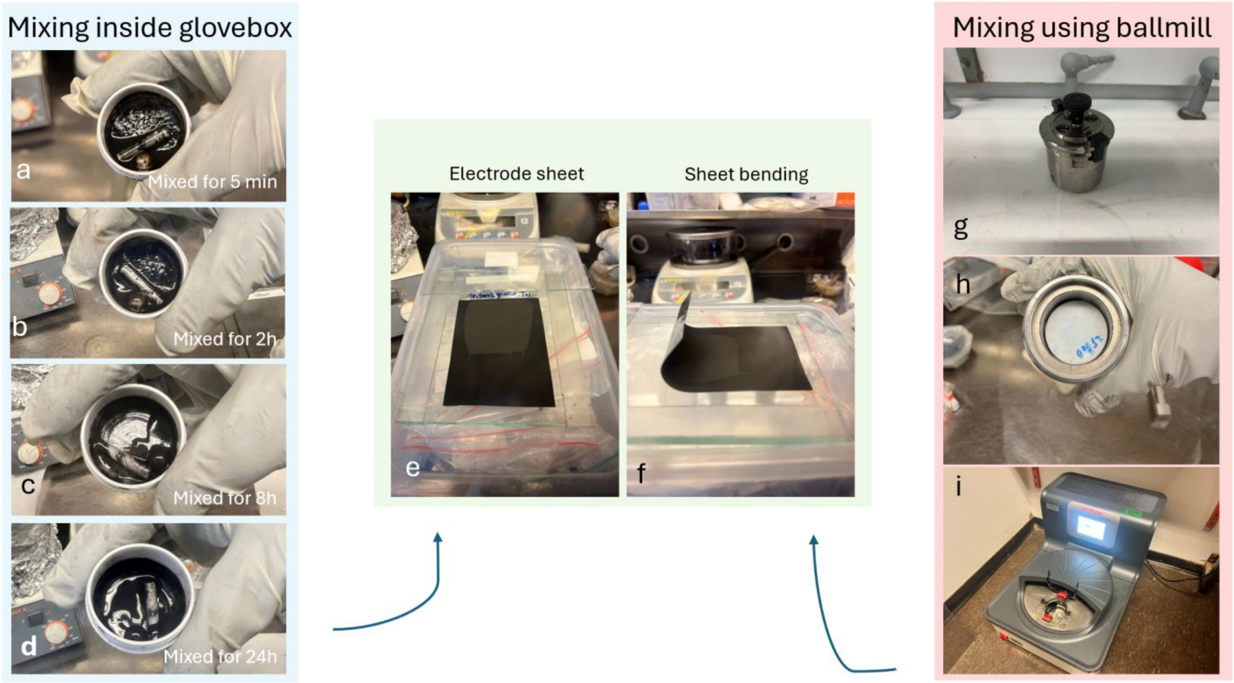

Figure S2: Illustration of two slurry making processes. (a, d): Slurry morphology mixed using magnetic stirrer inside glovebox at different time; (e-f): Electrode sheet, front and side views; (g-i) Slurry mixing using planetary ball mill.

Li_2FeSeO is air- and moisture-sensitive and thus needs to be treated in an inert atmosphere.[1, 2] Previous studies lack detailed description of the slurry mixing process. In this work, two simple approaches of slurry mixing were proposed: mixing using magnetic stirrer and mixing using ball mill.

For mixing using magnetic stirrer inside glovebox, Li_2FeSeO, polyvinylidene fluoride (PVDF), and carbon black in total of 200 mg with a mass ratio of 8:1:1 were pre-mixed and dissolved in 1.6 mL of NMP, the mixture, together with a stirring magnet and two zirconia balls were placed on a stirring plate. The rotation speed was set at 200 rpm, the slurry morphology evolution is shown in Figures S2a-2d. The slurry has a tendency of forming “jelly-like” agglomerates with insufficient mixing time, and roughly 24 h is needed to prepare a homogeneous slurry with adequate viscosity and density.

The same recipe was followed for mixing using a ball mill (Pulverisette 7) (Figures S2g-2i). The same container was placed inside a jar and wrapped with parafilm to maintain an inert atmosphere. The jar was then loaded into the ball mill, with the rotation speed set to 500 rpm. The process was carried out in cycles, each consisting of 20 minutes of milling followed by a 10-minute rest, for a total of 8 cycles.

The well-mixed slurry was coated on the aluminum foil (Figure S2e) using a 500 µm gapped stainless-steel rod. The foil was pre-coated with carbon to enhance the conductivity. Due to the

strong surface tension, the electrode sheet bends after drying overnight (Figure S2f). The electrode sheet was cut into 15-mm diameter electrodes and dried under vacuum at 120 ℃ for 5 h before coin cell assembly. The ball milled slurries were used for ex situ measurements while the slurries mixed using magnet were used for operando measurements and electrochemistry testing unless otherwise specified.

Section S2 Electrochemistry

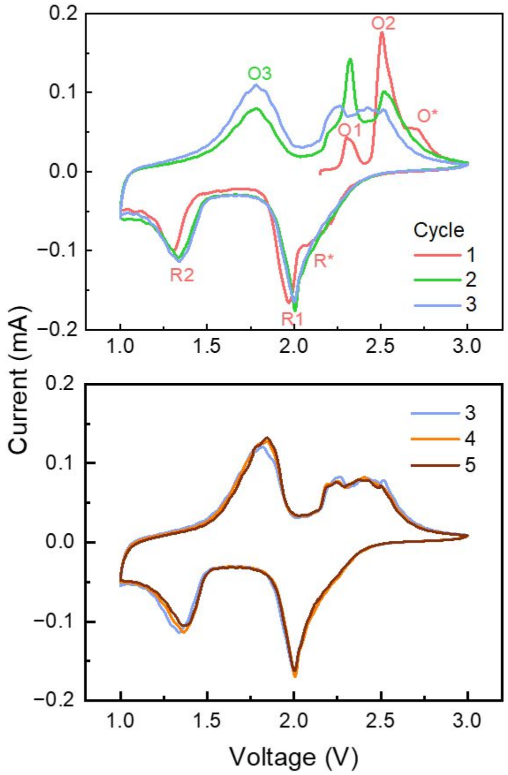

Figure S3: Comparison of CV profiles for the first three cycles and onwards.

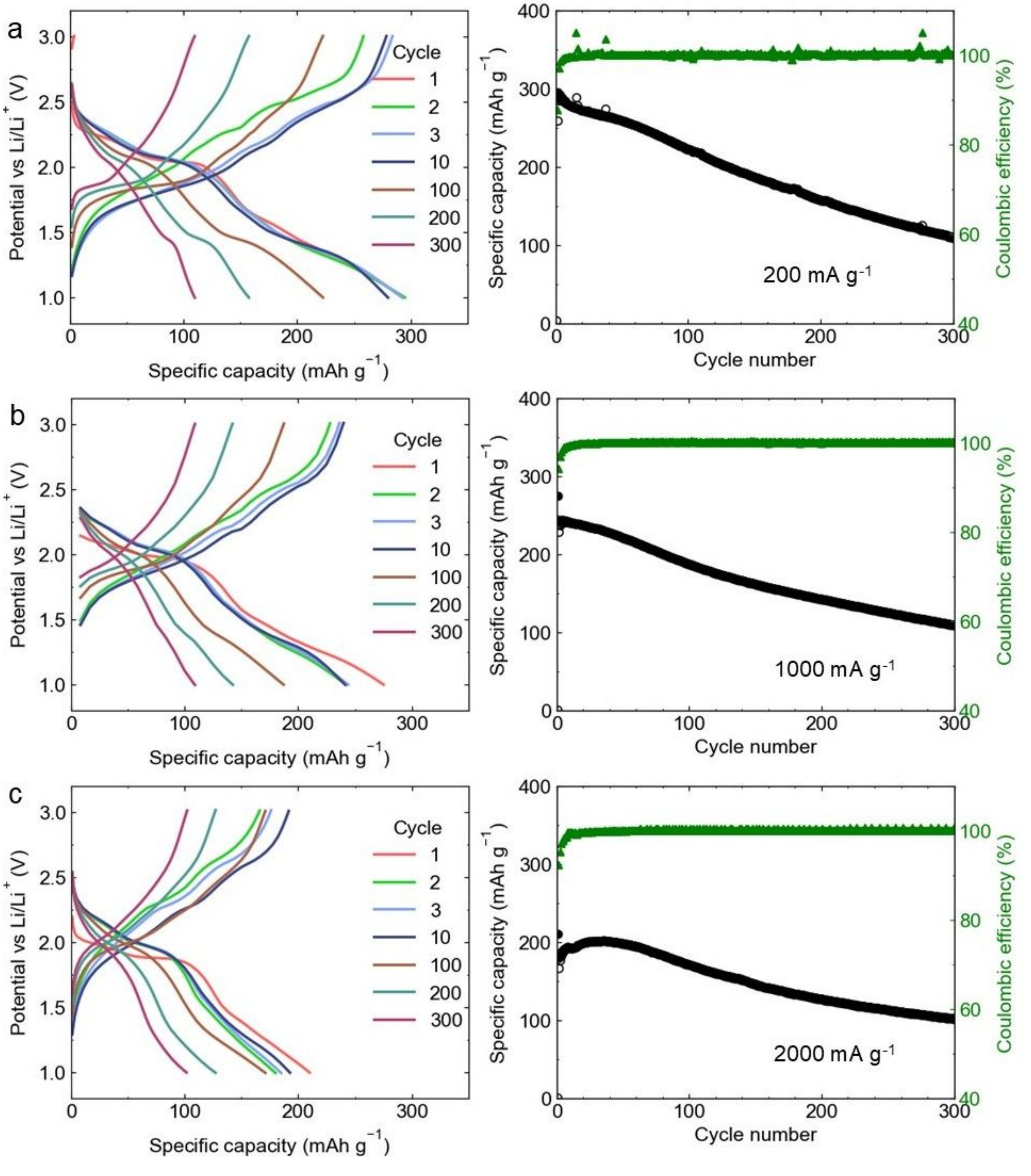

Figure S4: Rate performance of Li_2FeSeO. (a) 200 mA g^{-1}; (b) 1000 mA g^{-1} and (c) 2000 mA g^{-1}, with GC performance (left) and their corresponding CPC (right), respectively.

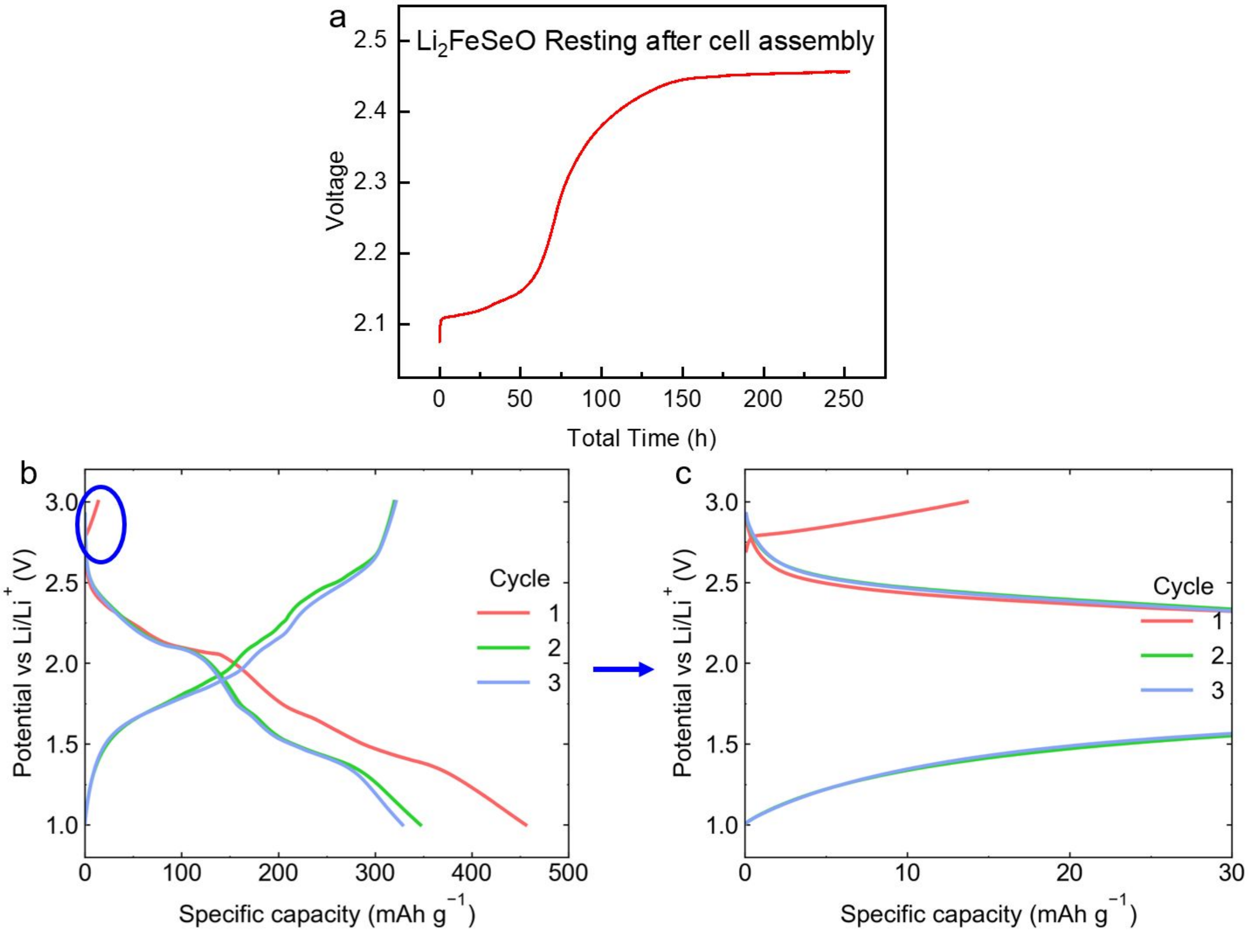

Figure S5: Relaxation of Li_2FeSeO cathode in the coin cell. (a) Relaxation of Li_2FeSeO coin cell; (b) GC curve of Li_2FeSeO after 30 days of coin cell assembly and (c): Zoom-in of (b).

The resting voltage profile (Figure S5a) of coin cell with Li_2FeSeO cathode indicates the phase transition that is similar to the electrochemistry of the first plateau, suggesting Li leaching out from the cathode,[3] resulting in increased voltage overtime. The leaching process slows down after the electrochemistry reaches the second plateau, however, the leaching process does not affect following cycles even at different rates.

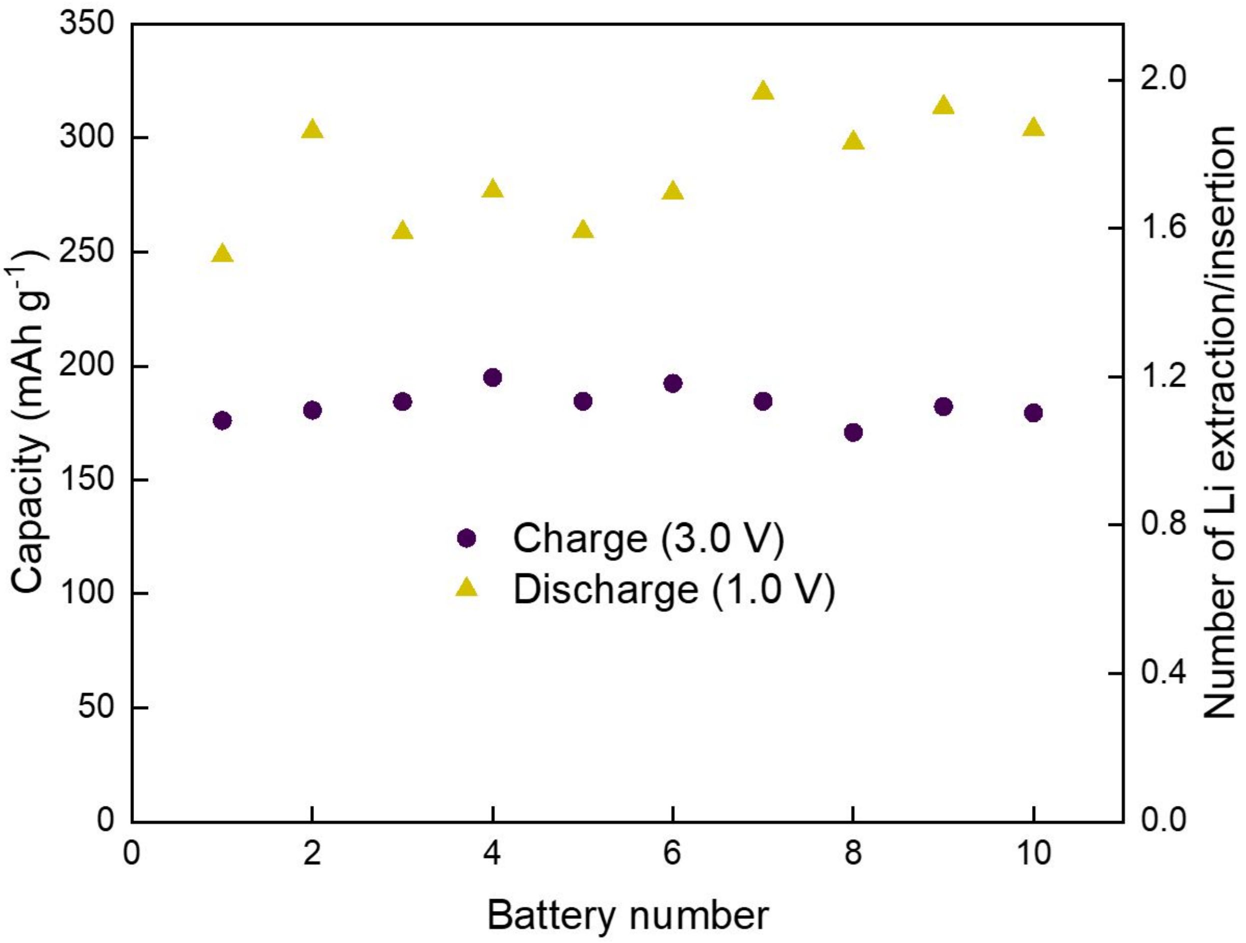

Figure S6: Capacity of the first charge and discharge at different stages of 10 batteries. The current density was 50 mA g^{-1}.

Figure S6 presents the capacity during the first charge at different stages. The left y-axis indicates the corresponding capacity, while the right y-axis represents the number of extracted Li^+ ions. During stage 1 (defined as the region between the starting point and the peak position of the first derivative of the GC profile), approximately 0.3 to 0.4 Li^+ per formula unit can be removed. By the end of stage 2 (3.0 V), a total of 1.1 Li^+ ions can be extracted. At the end of the first cycle, the structure allows for the reinsertion of 1.5 to 2 Li+ ions, corresponding to a discharge capacity of 250 – 310 mAh g^{-1}. Note that several factors could contribute to the uncertainties in the capacity value, including potential weighing errors of the electrodes and the inhomogeneities distribution of active material, binder and super P in the electrode sheet, resulting in an approximate 10% error in the measured capacity value. The slurries were mixed using ball mill shown in Figure S2.

Section S3 X-ray characterizations

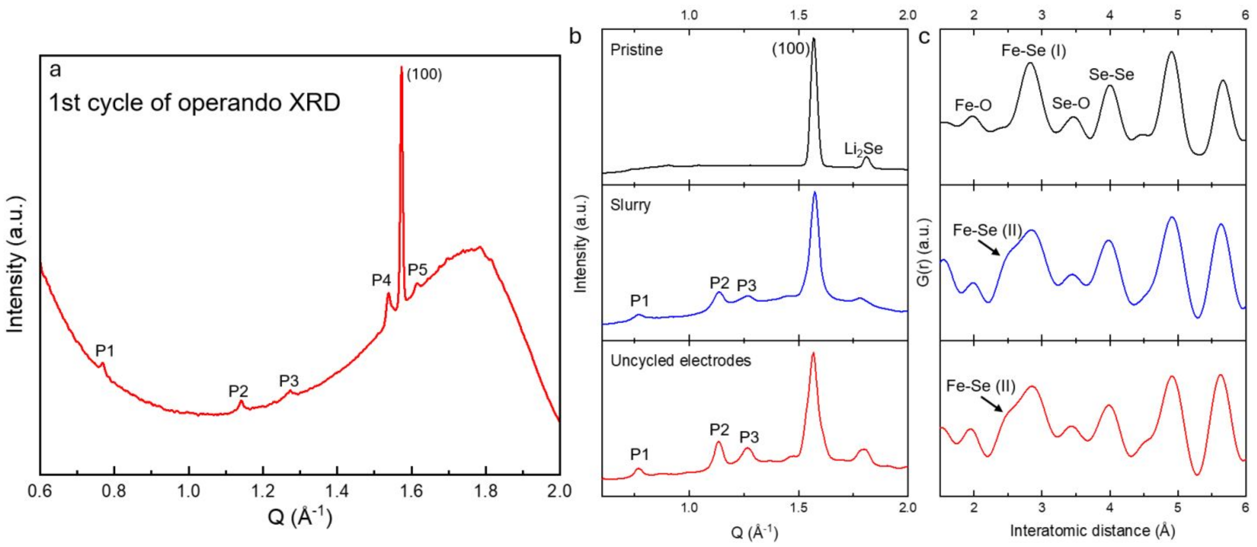

Figure S7: Tracking of structural change at different stages of cell assembly. (a) XRD of the 1st *operando* scan; (b) XRD at different stages of cell assembly and (c) corresponding PDF profiles in capillary mode. Data collected from BM31, ESRF.

As can be seen in *ex situ* and *operando* XRD, several peaks at low angle region were observed in Li_2FeSeO before cycling (Figures 3a and 3g), these peaks reflect superstructures of pristine Li_2FeSeO that possibility due to ordered vacancies of cation site. These peaks likely arise from small number of vacancies caused by Li^+ leaching during the slurry preparation and after cell assembly, which further leads to formation of superstructure. Three weak peaks shown in Figure 3a and Figure S7: P1 (0.768 Å^{-1}), P2 (1.140 Å^{-1}), and P3 (1.268 Å^{-1}) were observed during the first voltage plateau (2.2–2.4 V) of charge, evolving into P1* (0.790 Å^{-1}), P2* (1.225 Å^{-1}), and P3* (1.353 Å^{-1}) during the second voltage plateau (2.4–3.0 V). P1 is attributed to the (1/2, 0, 0) plane, P2 to the (1/2, 1/2, 0) plane, while P3 remains unidentified, but possibly corresponds to (1/2, 1/2, 1/2) plane. All peaks shifted to higher Q during charging indicating volume contraction and return during discharging.

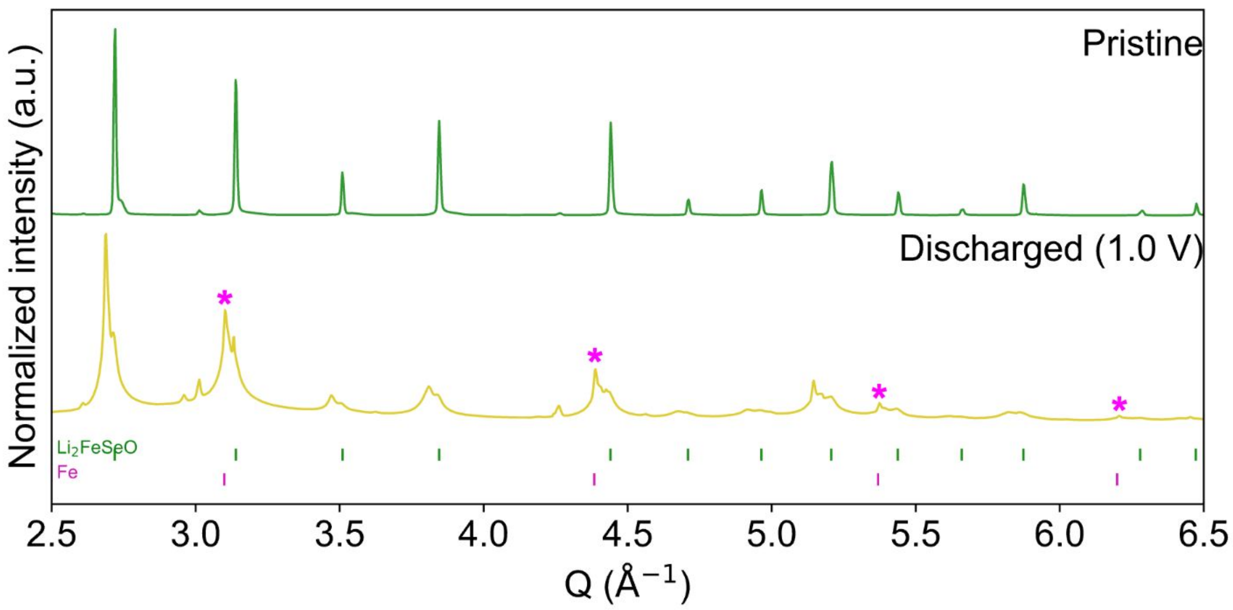

Figure S8: Fe peak indexing

As shown in Figure S8, which presents the *ex situ* measurement of the sample at the 1.0 V discharged state, all observed peaks are consistent with the Fe metal reference. However, the Fe peaks significantly overlap with those of the AP phase in the discharged state, as the AP lattice expands and all its peaks shift to lower-Q. This overlap makes a complete separation of the AP and Fe phases challenging. Nevertheless, the data indicate the possible formation of metallic Fe at the discharged state. Peaks corresponding to metallic Fe are also marked with asterisks in Figure 3g. For the PDF analysis, peak overlap is even more pronounced (Figure S8b), because the detector was positioned much closer to the sample that leads to broader peaks with lower resolution. This strong overlap may prevent clear resolution of the Fe–Fe distances, which may explain why metallic Fe is not directly observable in the PDF data.

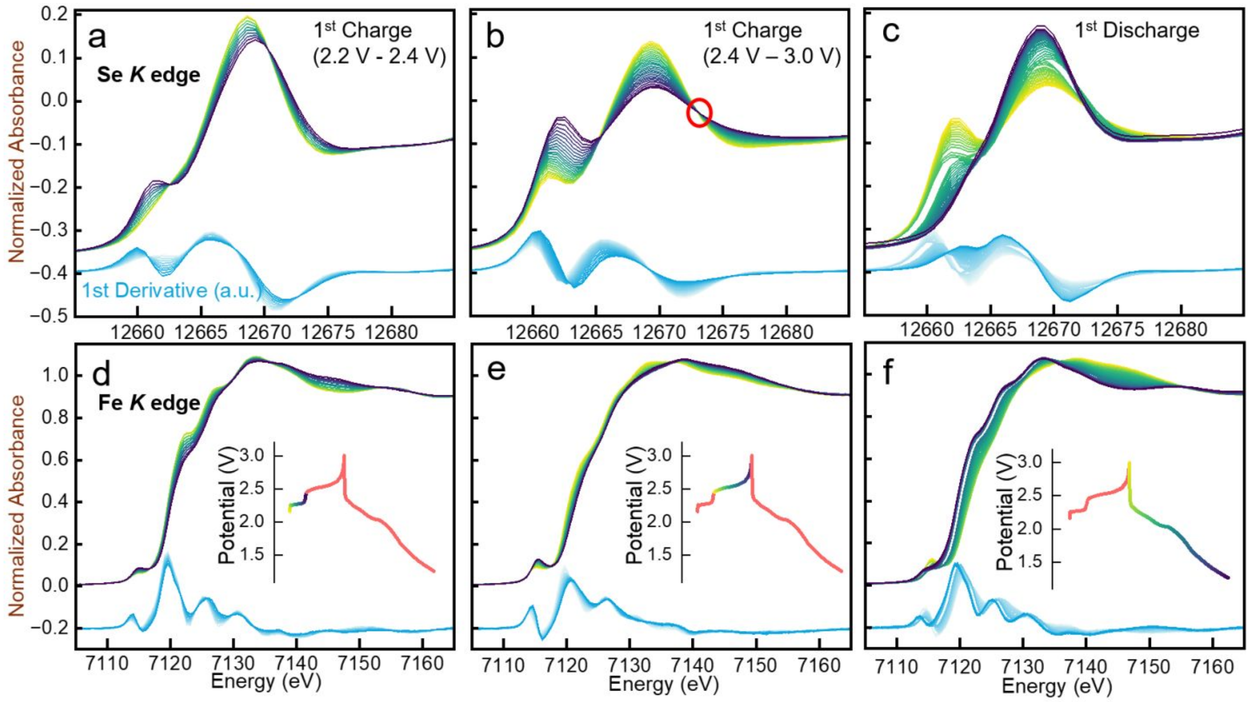

Figure S9: *Operando* XANES of Se and Fe *K*-edges. (a)-(c) Se *K*-edge and (d)-(f) Fe *K*-edge, respectively. The isosbestic point for Se *K*-edge during the second voltage plateau is marked with red circle indicating a bi-phasic structural change.

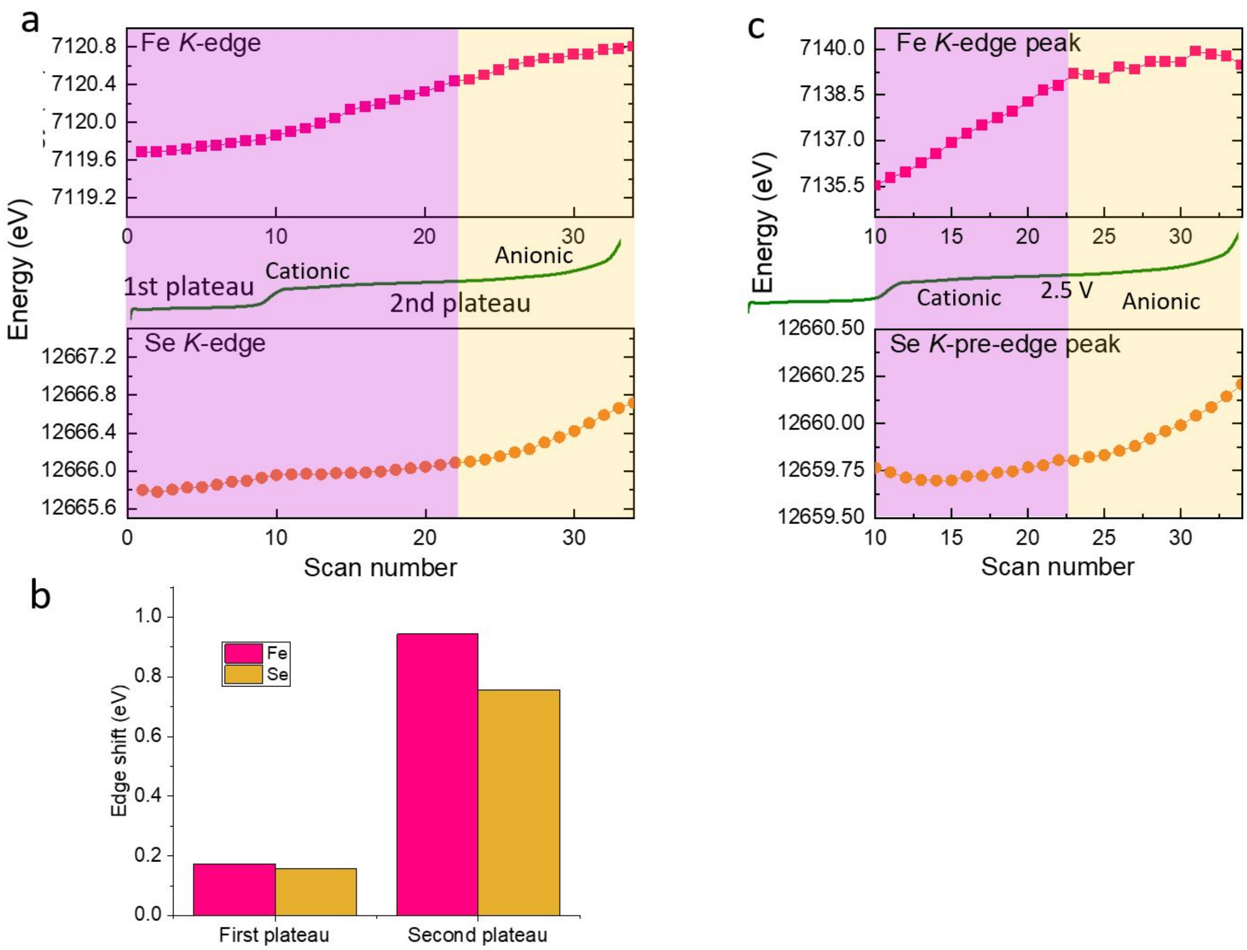

Figure S10: Comparison of redox behavior of Fe and Se during the first charge. (a) Comparison of main edge position of Fe and Se; (b) Comparison of the edge shift of Fe and Se during first and second voltage plateaus; (c) Comparison of Fe *K*-edge peak and Se *K*-pre-edge peak shift during the second voltage plateau.

To better illustrate the relative redox contributions of Se and Fe, a comparison of their edge behaviors is presented in Figure S10. It should be mentioned that a direct comparison of the average oxidation states of Se and Fe is challenging for the following reasons:

1. The reference compounds used for Se and Fe have coordination environments that differ from Se and Fe in AP structure, which limits the accuracy of direct oxidation state determination.
2. As shown in Figure S9, the redox behavior of Se and Fe differs significantly. During the first voltage plateau, Se *K*-edge shows a rise in pre-edge intensity, a shift of the main edge position to higher energy, and a decrease in main edge peak intensity, along with main edge peak shifting. In the meantime, Fe *K*-edge and peak gradually shift to higher energy. During the second voltage plateau, the redox behavior of Se changes. The pre-edge intensity continues to increase and shift, while the main edge intensity decreases. Although the main edge position also shifts to higher energy, this change arises mainly from a decrease in

edge peak intensity rather than a uniform shift. In contrast, Fe undergoes a more gradual edge and peak shift. The respective edge position shifts of Se and Fe K-edges are shown in Figures R6a and R6b. Where Fe has a stronger edge shift in both first and second voltage plateaus, but the trend slows at higher voltage, while Se edge shifts faster.

Directly comparing Se and Fe edge positions is complicated by their different edge shift mechanisms. While Fe overall exhibits a larger edge shift, most Se species form Se_2^{2-} pairs during the second plateau, favoring a Se^- state and indicating strong oxidation of Se in this period.

Nevertheless, for an intuitive comparison, instead of using edge positions, we compared the Fe edge peak position with the Se pre-edge peak position (Figure S10c). As the pre-edge feature of Se, arising from $1s \rightarrow 4p$ transitions, is more sensitive to changes in oxidation state than the main edge, which corresponds to $1s \rightarrow 5p$ transition. The determination of Se pre-edge peak position during the first plateau is difficult due to challenge of subtracting the base line and thus not provided. The comparison indicates that while the Fe edge peak shifts more significantly before 2.5 V during the second voltage plateau, its movement slows at higher voltage range (>2.5 V), whereas the Se pre-edge shift increases. A similar trend is seen in Figure S10a, where the Fe *K*-edge shift slows while the Se *K*-edge shift becomes more pronounced at higher voltages. These results are consistent with previous studies.[4] Although the exact oxidation state changes of Fe or Se cannot be quantified, there is clear evidence of increased Se participation in the redox process at voltages above 2.5 V.

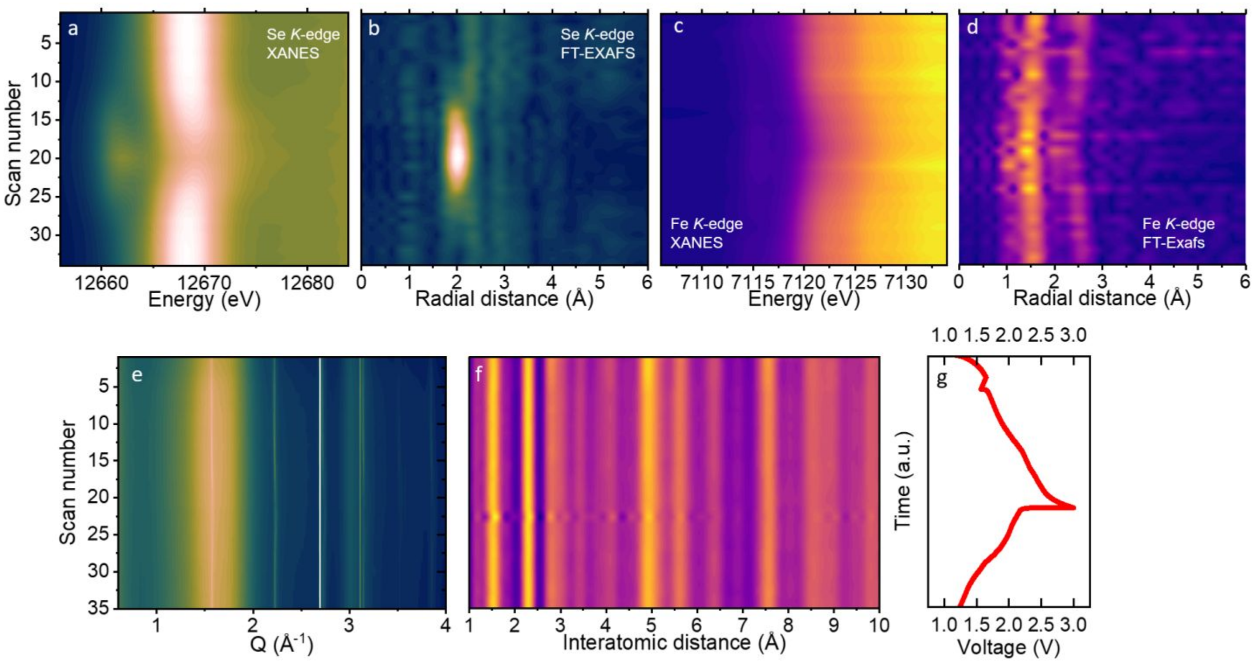

Figure S11: *Operando* XAS and XRD of Li_2FeSeO during the 2nd cycle. (a) *Operando* XANES of Se *K*-edge; (b) *Operando* FT-EXAFS of Se *K*-edge; (c) *Operando* XANES of Fe *K*edge; (d) *Operando* FT-EXAFS of Fe *K*-edge; (e) *Operando* XRD; (f) *Operando* PDF; (g) Corresponding GC profile.

Figure S11 shows the *operando* XAS and XRD of the second cycle. Similar to the first cycle, there is a reversible oxidation process of Se^{2-} to Se_2^{2-} with a shortening of Fe–Se distance that forms the Fe–Se_2O_2 tetrahedron at charged state (Figure S11b). *Operando* XANES of Fe *K*-edge shows a reversible redox reaction of Fe (Figure S11c), and a decreased Fe–Se distance that in accordance with Se FT-EXAFS. The increased background noise indicates an amorphization compared with the first cycle. Unlike the abrupt change in the Fe–Se bonding distance of the first cycle, the bonding distances change is more continuous at the second cycle. *Operando* XRD shows decreased peak intensities at charged state.

The corresponding operando XRD and PDF data are presented in Figures S11e and 11f. However, the interpretation of these results is complicated by a sudden rise in XRD background intensity, as illustrated in Figure S12.

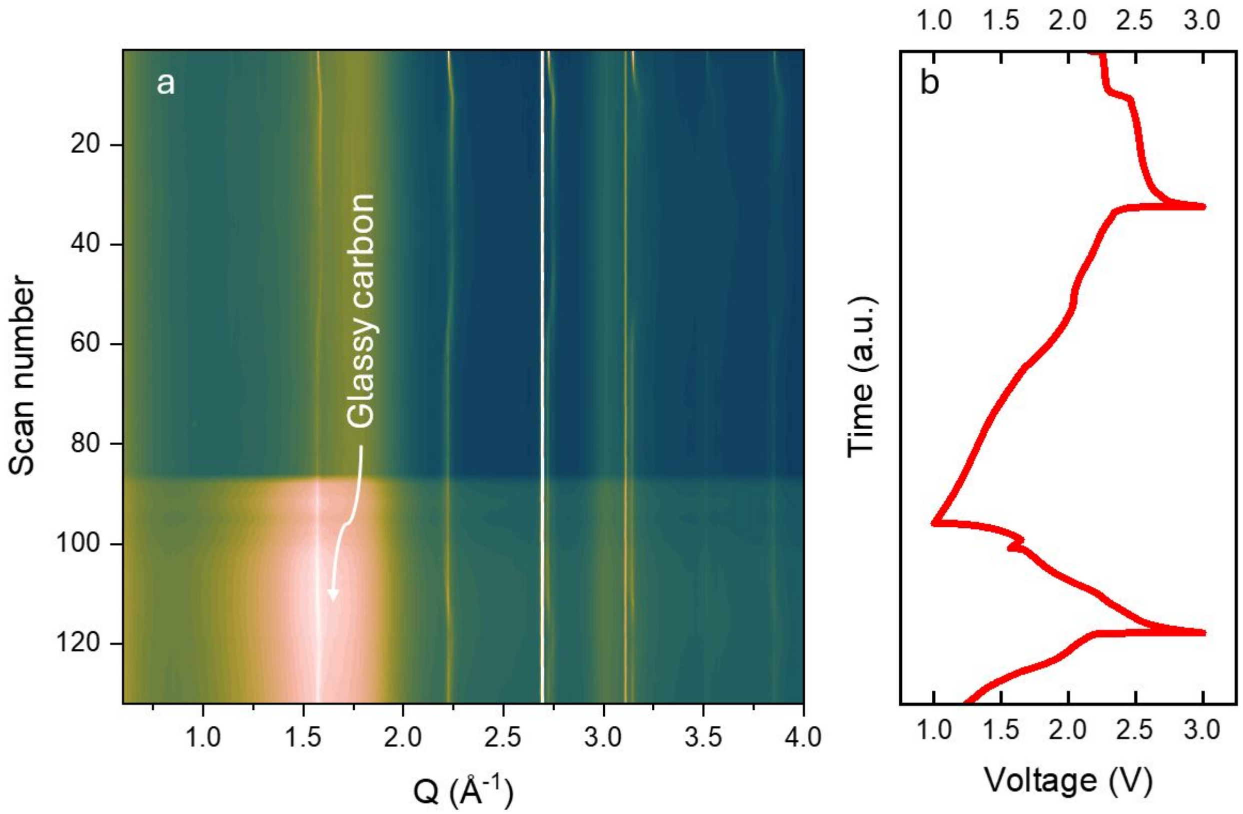

Figure S12: Increased XRD intensity upon lithiation.

It was found during the operando measurement in BM31, ESRF that the XRD peak intensity had a sudden increase upon lithiation below 1.25V. This sudden increase was accompanied with a strong disturbance of XAS signal (not shown).

One possible explanation is the intercalation of Li^+ into the glassy carbon window. As can be seen in Figure S10a, the bump in between 1.5-1.9 Å came from glassy carbon window, and the bump moved to left indicating an expanded glassy carbon unit cell volume. The voltage jump around 1.6V in Figure S12b is due to a short stop of the measurement for identifying the origin of the intensity change. The disturbance of XAS signal stopped during the second charge around 1.5V.

It was worth mentioning that *Operando* cells with both glassy carbon windows and with sapphire windows were measured in the beamline. It was found that glassy carbon windows have a stronger XRD background than sapphire windows, while less absorption which are more suitable for XAS study.

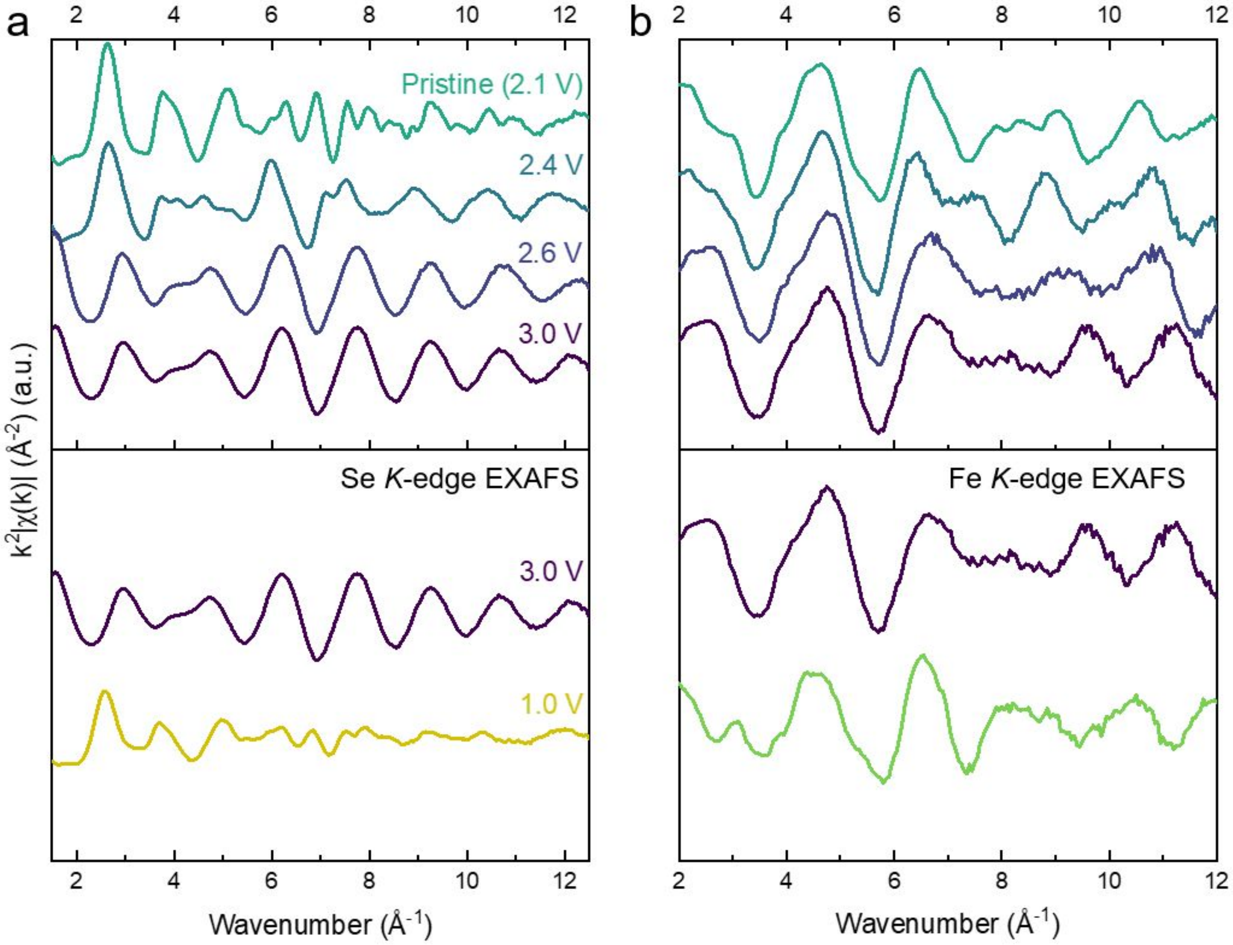

Figure S13: EXAFS of *ex situ* measurements of (a) Se and (b) Fe *K*-edges at different potential steps during the first cycle. The fitting range for Se was between 3 – 11 $Å^{-1}$, and for Fe was 2.5–12 $Å^{-1}$.

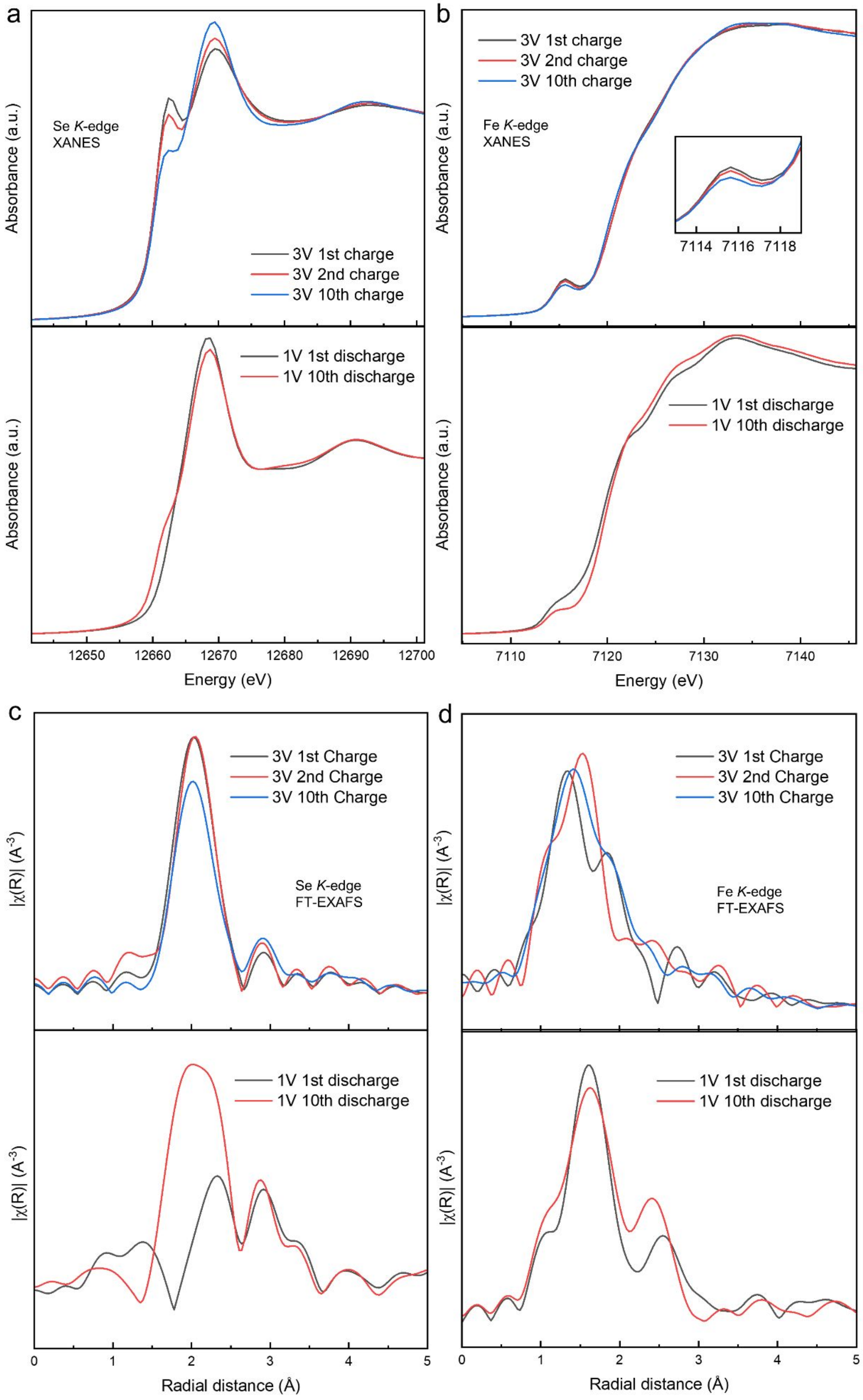
a
Se K-edge
XANES
3V 1st charge
3V 2nd charge
3V 10th charge
1V 1st discharge
1V 10th discharge
Absorbance (a.u.)
Energy (eV)
b
Fe K-edge
XANES
3V 1st charge
3V 2nd charge
3V 10th charge
1V 1st discharge
1V 10th discharge
Absorbance (a.u.)
Energy (eV)
c
Se K-edge
FT-EXAFS
3V 1st Charge
3V 2nd Charge
3V 10th Charge
1V 1st discharge
1V 10th discharge
|χ(R)| (A-3)
Radial distance (Å)
d
Fe K-edge
FT-EXAFS
3V 1st Charge
3V 2nd Charge
3V 10th Charge
1V 1st discharge
1V 10th discharge
|χ(R)| (A-3)
Radial distance (Å)

Figure S14: *Ex situ* XAS of Li_2FeSeO at different cycles. (a) and (e): Se *K*-edge XANES; (b) and (f): Fe *K*-edge XANES; (c) and (g): Se *K*-edge FT-EXAFS; (d) and (h) Fe *K*-edge FT-EXAFS.

Figure S14 shows the *ex situ* XANES and FT-EXAFS of Se and Fe *K*-edges at 1st, 2nd and 10th cycles. The pre-edge intensity of both Se and Fe decrease with increased cycle number at delithiated state, indicating an increased local disordering (Figures S14a and 14b). The presence of the strong peak in Se FT-EXAFS at 10th discharge (Figure S14c) and the absence of the same peak in Fe FT-EXAFS at 10th discharge (Figure S14d) indicates a new interaction other than Fe–Se (II) during lithiated state, which corresponds to perselenide Se_2^{2-}. The fitting range was 3–11 $Å^{-1}$ for both Se and Fe due to increased noise.

Table 1: Interatomic distances of Se and Fe coordinations (Å)

Atom	Se1	Fe1	O1	Se2	Fe2	O2
Se	4.0025	2.8302	3.4663	5.6604	4.9020	6.6374
Atom	Se1	Fe1	O1	Se2	Fe2	O2
Fe	2.8302	2.8302	2.00125	4.9020	4.0025	4.4749

Section S4 Fittings and calculations

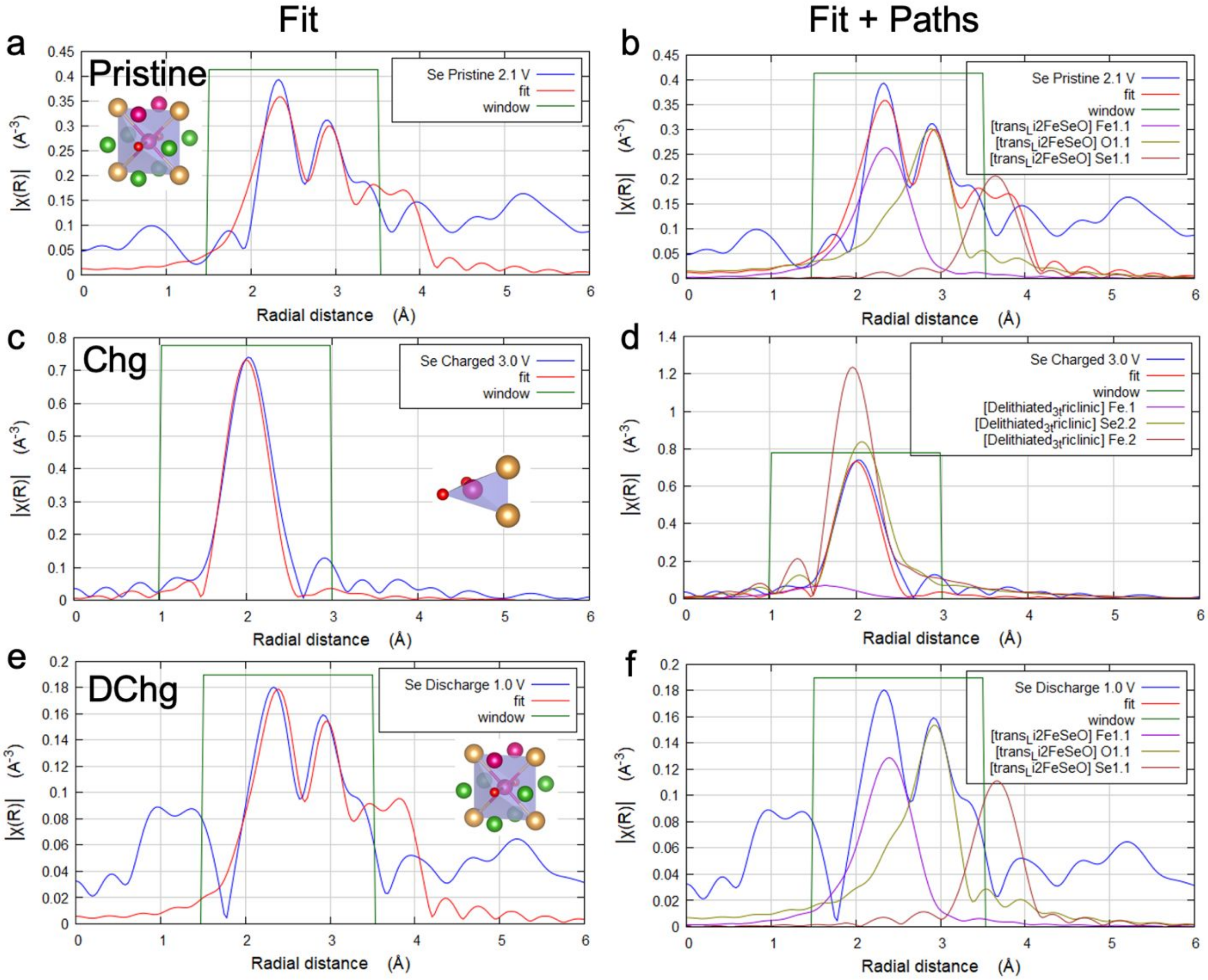

Figure S15: FT-EXAFS fitting of *ex situ* Se *K*-edge measurements. (a), (c), (e): Fitting results of pristine, charged (3.0 V) and discharged (1.0 V) states and (b), (d), (f) fitting results including individual path contributions, respectively.

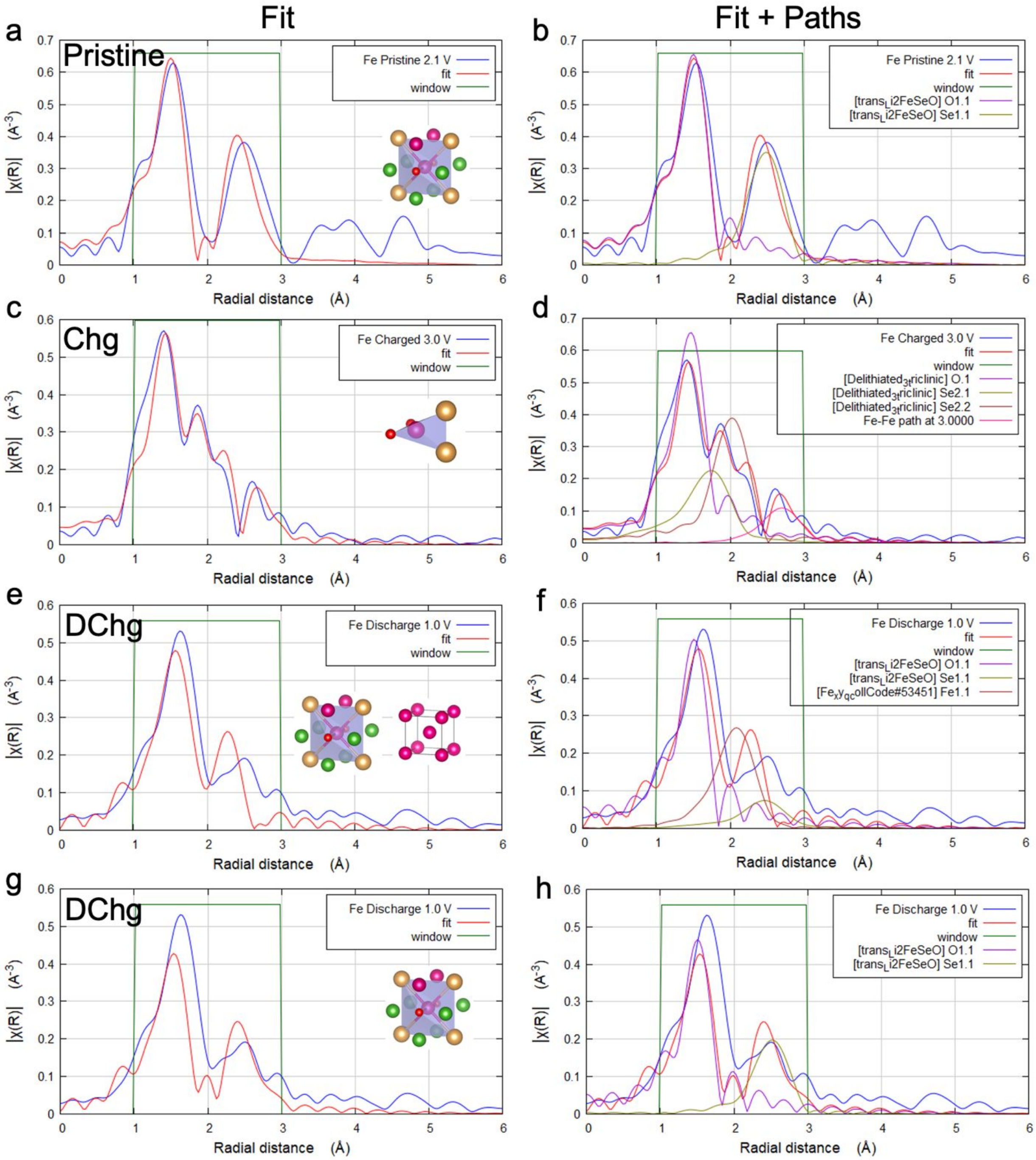

Figure S16: FT-EXAFS fitting of *ex situ* Fe *K*-edge measurements. (a), (c), (e), (g): Fitting results of pristine, charged (3.0 V), discharged (1.0 V) states (with and without Fe) and (b), (d), (f), (h) fitting results including individual path contributions, respectively.

EXAFS fitting based on Single Scattering (SS) was performed for both the pristine, charged and discharged structures. The total fit is shown in the left column and the contribution from each paths is shown in the right column.

For pristine state, as Artemis software does not support partial occupancy, a manually constructed local structure was used for the fitting with a trans-configuration. For the delithiated structure, the improved fit for both Se and Fe-*K* edges supports the formation of the proposed new local $FeSe_2O_2$ tetrahedral structure. For over-lithiated state, two phases were used for Fe *K*-edge, pristine AP and metal Fe. It should be noted that the fitting of 1.0 V Fe K-edge did not give a good match as fitting multiple phases (AP and metal Fe) using EXAFS is difficult, however, the contribution of each path does match well with the peaks observed in FT-EXAFS. The fitting parameters are shown in Table 2.

Table 2: EXAFS fitting parameters

Core atoms	Paths	N	$S0^2$	$Sigma^2$	E0	Delr	Reff	R
Se (Pristine)	Fe1	4	0.531	0.01718		-0.03726	2.83	2.79
	O1	8		0.00792		-0.04247	3.46	3.42
	Se1	6		0.01034		-0.01066	4.00	3.99
Se (Delithiated 3.0 V)	Fe1	1	0.948	0.03149		0.00000	2.16	2.16
	Se1	1		0.00039		0.00000	2.43	2.43
	Fe2	1		-0.00164		-0.14773	2.50	2.35
Se (Lithiated 1.0 V)	Fe1	4	0.263	0.01691	0	0.00039	2.83	2.83
	O1	8		0.00716		-0.00690	3.46	3.46
	Se1	6		0.00957		0.00603	4.00	4.00
Fe (Pristine)	O1	2	0.5	0.00113		-0.07121	2.00	1.93
	Se1	4		0.01104		-0.02832	2.83	2.80
Fe (Delithiated 3.0 V)	O1	2	0.596	0.00285		-0.27732	2.15	1.87
	Se1	2		0.01622		-0.08133	2.16	2.08
	Se2	2		0.00913		-0.14970	2.50	2.35
	Fe	2		0.01187		0.08241	3.00	3.08

	O1	2	0.316	-0.00091	-0.06985	2.00	1.93
Fit include metallic Fe	Se1	4	0.316	0.02193	-0.06022	2.83	2.77
	Fe	8	0.113	0.01158	0	2.48	2.48
Fe (Delithiated 1.0 V)	O	2	0.263	-0.00101	-0.07691	2.00	1.92
Fit without metallic Fe	Se	4	0.263	0.01026	0.00461	2.83	2.83

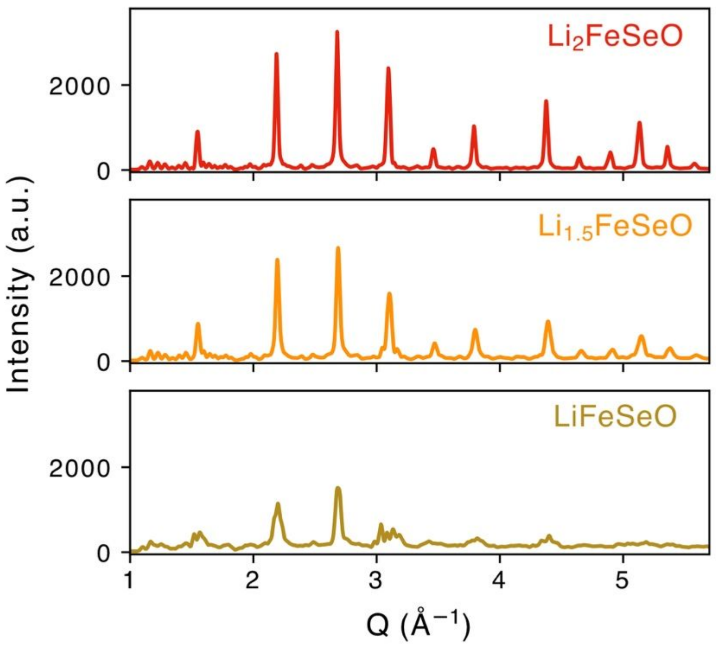

Figure S17: Simulated XRD. The structures calculated through DFT were used. To obtain the ensemble averages of XRD while considering the disordered occupancy of Li and Fe, we sampled five models of (4×4×4) supercells for each Li content and relaxed the structures.

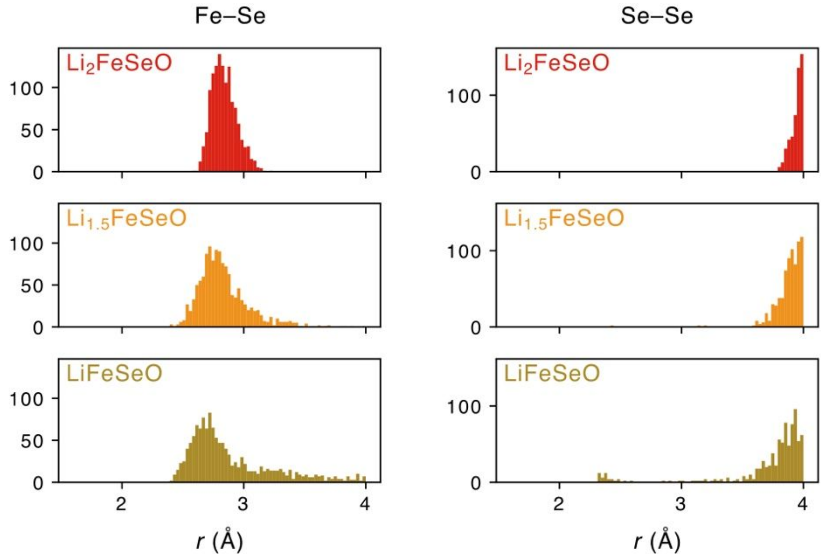

Figure S18: Simulated histogram of Fe–Se and Se–Se distances at different states of charge/discharge

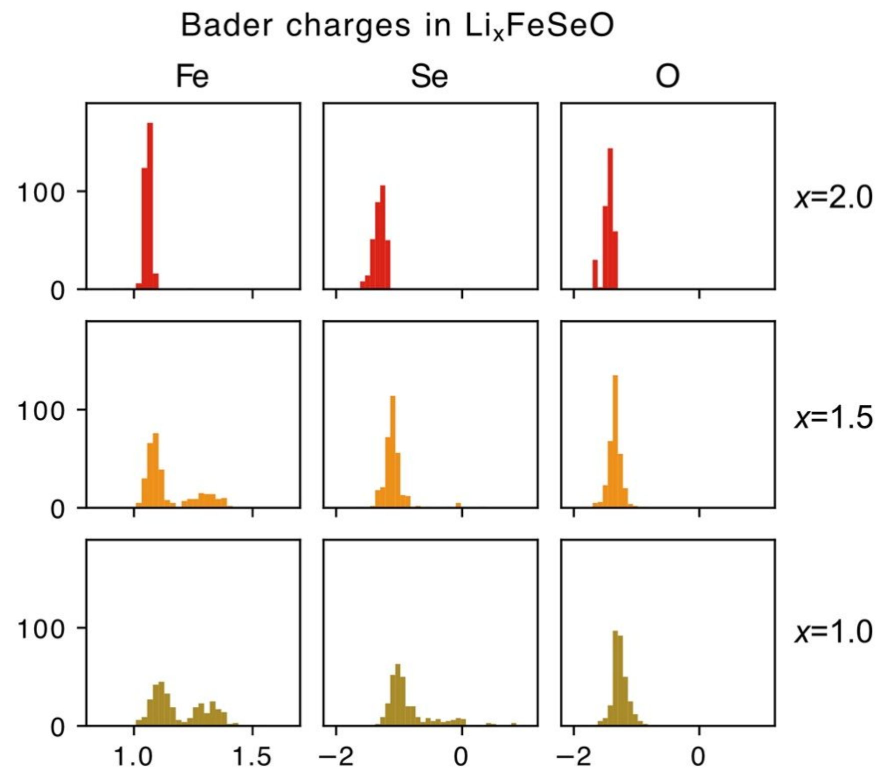

Figure S19: Simulated histogram of calculated Bader charge at different states of charge/discharge.

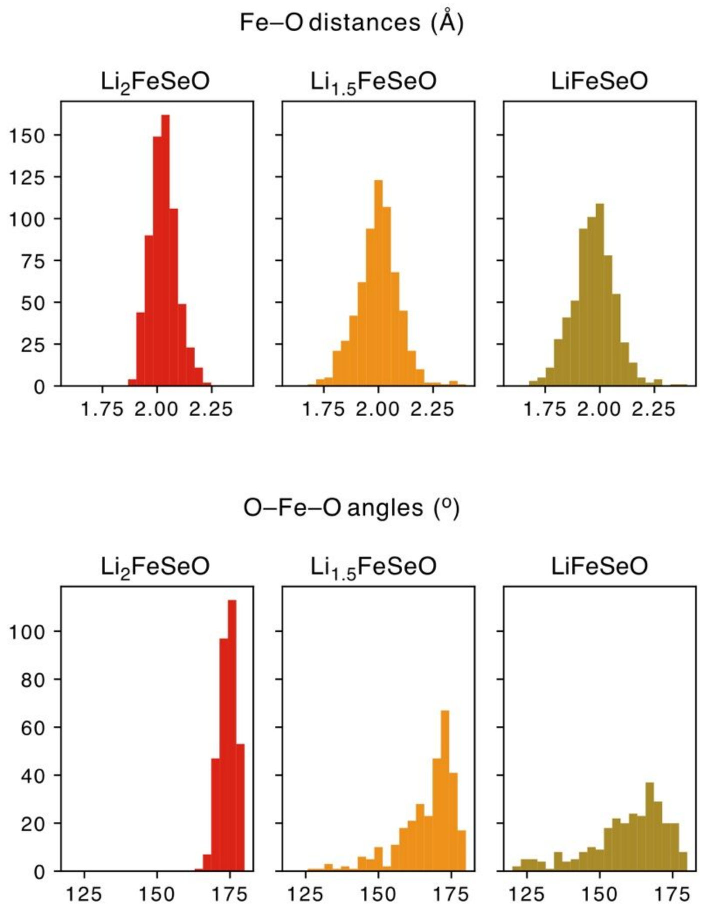

Figure S20: Simulated Fe–O distances and angles upon delithiation. The average distance of Fe–O bond decreases, similar to the results from EXAFS fitting, together with the decreased bond angles indicates changed –O–Fe–O– configuration from linear to zig-zag-like chian.

References
